# Amyloid β / PKC-dependent alterations in NMDA receptor composition are detected in early stages of Alzheimer´s disease

**DOI:** 10.1038/s41419-022-04687-y

**Published:** 2022-03-19

**Authors:** Carolina Ortiz-Sanz, Uxue Balantzategi, Tania Quintela-López, Asier Ruiz, Celia Luchena, Jone Zuazo-Ibarra, Estibaliz Capetillo-Zarate, Carlos Matute, José L. Zugaza, Elena Alberdi

**Affiliations:** 1 Department of Neuroscience, University of Basque Country (UPV/EHU) and CIBERNED, Leioa, Spain; 2 Achucarro Basque Center for Neuroscience, Leioa, Spain; 3 IKERBASQUE Basque Foundation for Science, Bilbao, Spain; 4 Department of Genetics, Physical Anthropology and Animal Physiology, UPV/EHU, Leioa, Spain; 5 Present Address: Department of Neuroscience, Physiology, & Pharmacology, University College London, London, UK

**Keywords:** Alzheimer's disease, Extracellular signalling molecules

## Abstract

Amyloid beta (Aβ)-mediated synapse dysfunction is an early event in Alzheimer’s disease (AD) pathogenesis and previous studies suggest that NMDA receptor (NMDAR) dysregulation may contribute to these pathological effects. Although Aβ peptides impair NMDAR expression and activity, the mechanisms mediating these alterations in the early stages of AD are unclear. Here, we observed that NMDAR subunit NR2B and PSD-95 levels were aberrantly upregulated and correlated with Aβ_42_ load in human postsynaptic fractions of the prefrontal cortex in early stages of AD patients, as well as in the hippocampus of 3xTg-AD mice. Importantly, NR2B and PSD95 dysregulation was revealed by an increased expression of both proteins in Aβ-injected mouse hippocampi. In cultured neurons, Aβ oligomers increased the NR2B-containing NMDAR density in neuronal membranes and the NMDA-induced intracellular Ca^2+^ increase, in addition to colocalization in dendrites of NR2B subunit and PSD95. Mechanistically, Aβ oligomers required integrin β1 to promote synaptic location and function of NR2B-containing NMDARs and PSD95 by phosphorylation through classic PKCs. These results provide evidence that Aβ oligomers modify the contribution of NR2B to NMDAR composition and function in the early stages of AD through an integrin β1 and PKC-dependent pathway. These data reveal a novel role of Aβ oligomers in synaptic dysfunction that may be relevant to early-stage AD pathogenesis.

## Background

Aβ oligomers accumulate both in AD patients and animal model brain tissue [1, 2] and are the most toxic and pathogenic form of Aβ peptide [3]. Aβ oligomers bind to receptors in order to activate particular aberrant cellular responses [4], which are critically involved with tau pathology [5], inflammation, oxidative stress and synapse loss and dysfunction [6].

NMDARs are ionotropic glutamate receptors that actively control synaptic plasticity and synapse formation, and are responsible for memory function, learning and formation of neuronal networks in the central nervous system (CNS) [7]. Aβ peptides increase intracellular calcium and ROS production through NMDAR-dependent mechanisms [8, 9], and initiate the impairment of NMDAR activity by regulation of protein levels and localization of NMDAR subunits [10]. Additionally, channel properties of NMDARs are regulated by Protein Kinase C family members (PKCs), which phosphorylate NMDAR subunits in specific serine/threonine residues [11]. Important molecular events in Aβ oligomer pathology are regulated by PKC signaling pathways [12, 13]. Gain-of-function mutations in the gene encoding PKCα were described in some families with inherited Alzheimer’s disease, thus enhanced PKCα activity may contribute to synaptic deficits in AD [14, 15].

NMDA receptor surface trafficking and composition are developmentally regulated by extracellular matrix proteins requiring β1-containing integrin receptors [16]. Moreover, synaptic integrins activate local protein kinases which phosphorylate and modulate NR2A and NR2B subunits of NMDARs, enhancing NMDAR-mediated function in mature hippocampal synapses [17]. Integrins are a large family of extracellular matrix receptors ubiquitously expressed and distributed and previous data pointed out them as candidates for Aβ peptide receptors in astrocytes, neurons and oligodendrocytes [18–20].

In this study, we first investigated early synaptic changes in human AD brains. We found that, in fractions enriched in the postsynaptic density from AD patients, NR2B subunit of NMDA receptor and scaffolding protein PSD-95 were overexpressed in early stages of the disease and positively correlated with Aβ peptide load. Accordingly, similar results were found in the 6-month-old 3xTg-AD mouse hippocampi and in Aβ-injected mouse brains. Finally, we demonstrated in vitro that Aβ oligomers differentially increased the NR2B-containing NMDAR and PSD-95 and enhanced NMDAR-dependent Ca^2+^ influx by mechanisms involving NR2B subunit phosphorylation in an integrin β1 / PKC signaling pathways. Overall, these results demonstrated that dysregulation of NMDARs in early stages of AD might be provoke by Aβ oligomers enhancing an aberrant calcium function by altered localization of NR2B-containing NMDARs in an integrin β1 and PKC-dependent manner.

## Results

### NR2B subunit and PSD95 levels increase in postsynaptic density fractions of prefrontal cortex from early-stage AD patients and correlate with Aβ levels

**Fig. 1:**
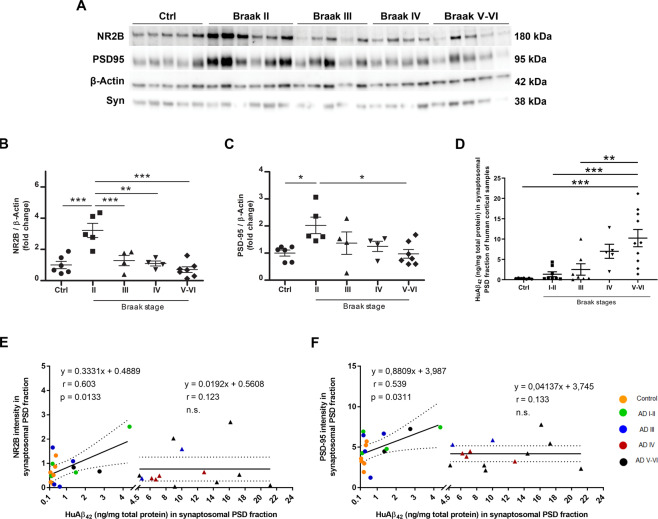
NR2B and PSD-95 levels are elevated in prefrontal cortex from AD patients at early disease stages, and correlate with Aβ load. **A** Western blot of NR2B, PSD-95 and β-actin in PSD fraction of *post-mortem* prefrontal cortex from controls and AD patients at different Braak stages (Braak II-VI) (Supplemental Material). **B–C** Scatter plot showing NR2B and PSD-95 levels in controls (Ctrl) and AD subjects (*n* = 4–7 per group). Data were analysed with one-way ANOVA followed by Bonferroni´s test; *p < 0.05, **p < 0.01, ***p < 0.001. (D) ELISA determination of Aβ peptide levels in PSD fractions of samples with different AD stages and controls as indicated in the scatter plot. Data were analysed with one-way ANOVA followed by Sidak´s test; **p < 0.01, ***p < 0.001. **E, F** Scatter plots of NR2B and PSD95 versus Aβ levels in samples of PSD fractions of human prefrontal cortex from controls and AD patients. Color codes of control and AD samples are indicated in F. Note the significant positive correlation between NR2B (*n* = 26 samples) or PSD95 (*n* = 29 samples) and Aβ levels in control and AD I-III samples (*p* = 0.0133 and *p* = 0.0311, respectively). Data were analyzed with a linear regression method.

Reorganization and distribution of NMDA receptor subunits and postsynaptic protein PSD-95 occur in AD brains [21]. To identify changes during the progression of disease, we analyzed the PSD and non-PSD enriched fraction of isolated synaptic terminals purified from the prefrontal cortex of twenty-six samples of nondemented controls *n* = 6 and AD brain patients, classified as AD-II *n* = 5, AD-III *n* = 4, AD-IV *n* = 4 and AD-V-VI *n* = 7 (supplementary Table 1) [22]. Western blot analysis of NR2B subunit (Fig. 1A, B) and PSD95 (Fig. 1A, C) revealed a significant increase of both proteins in PSD fractions particularly at Braak II stage, compared to nondemented control group and to Braak III, IV, and V-VI classified subjects. Expression of both proteins decreased concomitantly from Braak II to Braak VI stages, which reveals a significant transient increase in the early stages of the disease, followed by a decrease during disease progression (Fig. 1A–C). Accordingly, synaptophysin levels were also increased specifically in non-PSD fractions of Braak II stage AD brains when compared to controls and Braak V-VI (supplementary Fig 1). In addition, quantification of Aβ_42_ by ELISA (n = 37 samples, supplementary Table 1) in PSD extracts showed increasing levels throughout AD stages (Fig. 1D). Importantly, Aβ_42_ levels in PSD fractions ranged from 0.1 to 4.5 ng/mg protein and positively correlated with synaptic proteins in human samples (Fig. 1E, F). However, when Aβ_42_ levels exceeded 4.5 ng/mg protein, these correlations were lost, pointing out towards an Aβ_42_ threshold concentration for NR2B and PSD95 upregulation. Taken together, these data show a specific increase of NR2B subunit and PSD95 that correlates with Aβ_42_ levels in the prefrontal cortex of brain samples at early stages of AD, which is counteracted along the progression of disease. These important changes suggest a critical temporal dynamics of molecular reorganization of the synapse throughout the course of the disease that is dependent on Aβ_42_ peptide.

### Aβ peptide levels correlate with NR2B and PSD95 deregulation in the 3xTg-AD mice

**Fig. 2:**
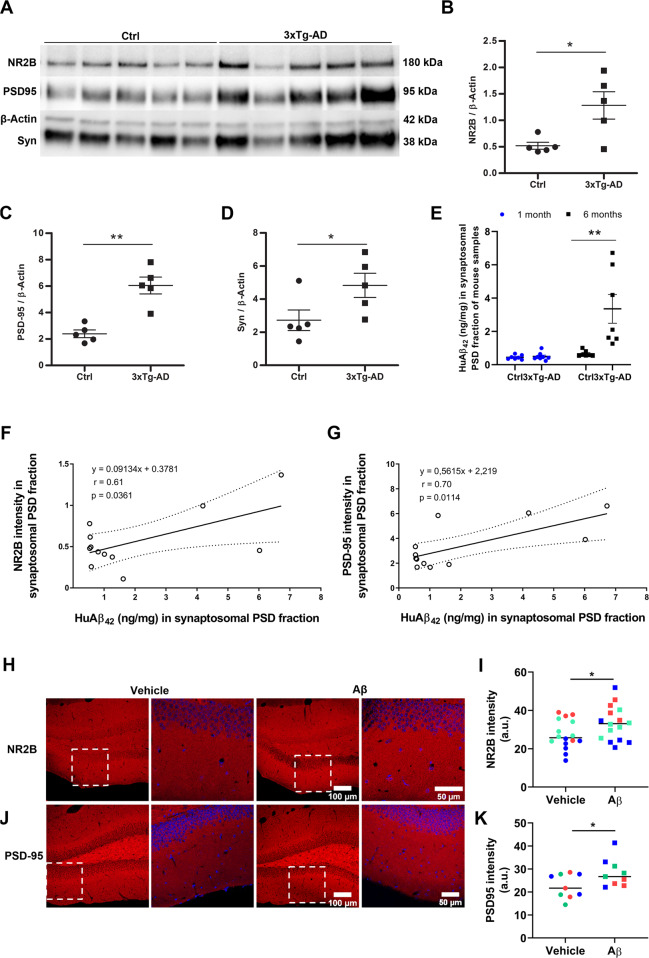
Aβ peptide load in 3xTg-AD mice correlates with NR2B and PSD95. **A–D** Western blots and quantitative analysis of NR2B, PSD-95, and synaptophysin (Syn) in isolated synaptic terminals of 6-month-old control and 3xTg-AD mice (*n* = 5 animals per group) (Supplemental Material). Scatter plots represent the means ± S.E.M. of values normalized to corresponding β-Actin; *p < 0.05, **p < 0.01; unpaired Student’s *t* test. **E** Scatter plot of Aβ peptide levels in synaptosomes of same samples as determined by ELISA. Data were analyzed with two-way ANOVA test followed by Sidak´s multiple comparison test; **p < 0.01. **F, G** Scatter plots of NR2B and PSD95 versus Aβ levels in synaptosomes of controls and 3xTg-AD mice. Note the significant positive correlation between NR2B or PSD95 and Aβ levels in mouse samples (*p* = 0.036 and *p* = 0.011, respectively). Data were analyzed with a linear regression method. **H, J** Coronal sections of mouse brains were analyzed after 7 days of vehicle or Aβ (135 ng) injection. Photomicrographs show NR2B **(H)** and PSD-95 **(J)** immunolabeling in dentate gyrus. **I, K** Scatter dot plots show the mean values of NR2B and PSD95 intensities in vehicle- and Aβ-injected mice. 2–3 brain sections of 9–16 mice were used. Different experiments are represented by colors, and dots are data obtained in each animal. Data were analyzed with unpaired Student’s *t*-test; *p < 0.05.

To study molecular distribution and organization of NR2B, PSD95, synaptophysin, and Aβ_42_ along synaptic deficit progression, we initially selected an age with no signs of pathology in the triple transgenic mouse model (3xTg-AD) (1 month; Fig. 2E; supplemental Fig S2) and another when Aβ is just emerging (6 months; Fig. 2) [23]. Isolated synaptosomes from the hippocampus were analyzed by ELISA and Western blot. In 6-month-old 3xTg-AD mice, we observed a significant increase in NR2B subunit (Fig. 2A, B; 1.28 ± 0.29 vs. 0.52 ± 0.07 arbitrary units, a.u.), PSD-95 (Fig. 2A, C; 5.83 ± 2.81 vs. 2.39 ± 0.29 a.u), synaptophysin (Fig. 2A, D, 4.83 ± 0.73 vs. 2.72 ± 0.62 a.u) and Aβ42 levels (Fig. 2E; 3.35 ± 0.86 vs. 0.66 ± 0.07 a.u) as compared to control animals (*n* = 5 mice in all instances). Notably, these data showed a robust correlation between Aβ42 with NR2B (*p* = 0.036) and PSD95 (*p* = 0.011) (Fig. 2F, G). As control, protein levels of 1-month old mouse showed similar expression in 3xTg-AD and WT mice (Supplementary Fig 2, *n* = 7 and 8 mice, respectively). Thereby, these results indicate that 6-month-old 3xTg-AD mice show an enhanced expression of NR2B subunit, PSD-95 and synaptophysin proteins, which is associated with increased levels of Aβ42 peptides in isolated synaptic terminals of the hippocampus.

### Aβ injection increases NR2B and PSD95 levels in adult mice hippocampus

To elucidate the biological effects of Aβ oligomers in NR2B and PSD95 protein expression, we injected Aβ oligomers or vehicle into the hippocampus of C57 adult mice. Aβ injection led neurons to overexpress NR2B and PSD95 proteins in DG, as revealed by quantification of the immunolabeled area with synaptic markers NR2B (Fig. 2H, I; 33.3 ± 2.2 vs. 27.3 ± 2.0 a.u; Aβ- vs. vehicle-injected mice (*n* = 16 animals, in three experiments) and PSD-95 (Fig. 2J, K; 28.2 ± 2 vs. 22.5 ± 1.7 a.u; Aβ- vs vehicle-injected mice (*n* = 9 animals, in three experiments). However, any change in NR2B and PSD95 expression was observed in CA1 and CA3 areas (Supplemental Figure S3 and S4, respectively). Overall, these histological findings demonstrate that Aβ oligomers trigger NR2B and PSD95 upregulation in DG of mouse hippocampus.

### Aβ oligomers regulate NR2B-containing NMDA receptor density and related Ca^2+^ response in neuronal membranes

**Fig. 3:**
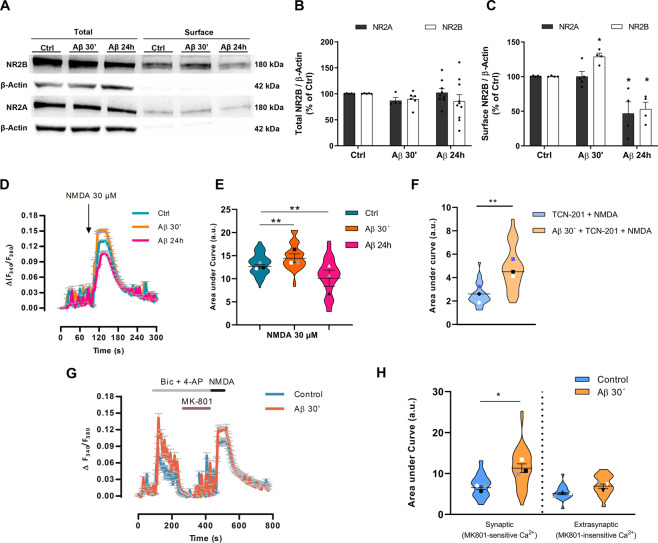
Aβ promotes alterations of NR2A and NR2B subunit distribution and function in neurons. **A–C** Cultured neurons were incubated with Αβ 1 µM for 30 min or 24 h, and total and biotinylated neuron-surface NR2A and NR2B were identified by western blot (**A,** supplemental material). Graph bars (means ± S.E.M.) represent the volume band intensities of total NMDAR subunits (**B**) and surface NMDA receptors (**C**) normalized to β-actin and expressed as a percentage of untreated cells. *p < 0.05, compared to non-treated (control) cells; paired two-way ANOVA. **D, E** Neurons were exposed to 1 µM Aβ for 30 minutes or 24 h, loaded with Fura-2AM for 30 minutes and Ca^2+^ rise was evaluated after application of 30 µM NMDA, 1 min. Each recording represent the average of 52–65 cells from 3 independent experiments. **E** Violin plot represents data distribution and mean ± S.E.M. of the area under curve for each condition in arbitrary units (a.u.). Data were analyzed with one-way ANOVA followed with Dunnet´s test; **p < 0.01; *n* = 3 cultures, 191 cells in total analysis. **F** Effects of 10 µM TCN-201, an NR2A antagonist, in NMDA-induced Ca^2+^ influx (1 min) in untreated and Aβ-treated neurons for 30 min. Violin plot shows data distribution and mean ± S.E.M. of area under curve of NMDA responses. Data were analyzed with two-tailed unpaired Student’s *t*-test; **p < 0.01. **G** Ca^2+^ recordings following synaptic and extrasynaptic NMDAR activation in control and neurons treated with 1 µM Aβ for 30 min (*n* = 3 experiments; 67 and 63 cells, respectively). Synaptic NMDAR activity was recorded after addition of 50 µM bicuculline and 2.5 mM 4-AP. Extrasynaptic NMDAR-mediated ion fluxes were recorded with NMDA agonist (30 µM) following synaptic NMDAR blockade with 10 µM MK-801 applied during treatment with bicuculline. **H** Violin plot represents data distribution and mean ± S.E.M. of area under Ca^2+^ response curve for control (untreated-cells) and Aβ-pretreated cells in synaptic and extrasynaptic-associated recorders. Data were analyzed with unpaired Student’s *t*-test; *p < 0.05.

Cultured neurons were treated with 1μM Aβ for 30 min or 24 h, and cell surface receptor density was determined by cleavable biotinylation assay followed by SDS-PAGE and Western blotting. As shown in Fig. 3A, C, Aβ acute treatment for 30 min enhanced NR2B but not NR2A subunits at neuronal membrane surfaces (129.8 ± 7.6% compared to control vehicle-treated cells 100%, *n* = 4 cultures). Conversely, longer Aβ treatment for 24 h reduced both NR2A and NR2B subunit localization at cellular surface (46.9 ± 16.6% and 53.2 ± 9.8%, respectively, compared with control vehicle-treated cells, 100%; Fig. 3A, C; *n* = 4 cultures). Neither acute nor chronic Aβ treatments exerted significant effect on the expression of NR2A or NR2B subunits in total cell lysates (Fig. 3A, B; *n* = 6–10). In addition, although Aβ treatment increased both cytosolic [Ca^2+^] ([Ca^2+^]_cyt_) and the presence of NR2B in the neuronal membrane, excitotoxicity elicited by the NMDA post-treatment was not significantly affected with respect to untreated cells (Supplemental Figure S5). These results show that Aβ selectively modulates the membrane localization of NR2A and NR2B subunits in NMDA receptors of primary cortical neurons.

Next, [Ca^2+^]_cyt_ induced by NMDA was recorded in neurons pretreated with 1 μM Aβ for 30 min or 24 h. Aβ-pretreatment for 30 min significantly enhanced the [Ca^2+^]_cyt_ after NMDA application, while chronic pretreatment with Aβ reduced the NMDA-induced Ca^2+^ response compared to control (Fig. 3D, E, 12.7 ± 0.3, 14.6 ± 0.4 and 10.8 ± 0.5 a.u., respectively; *n* = 3 cultures, 191 cells). Furthermore, NMDA-dependent [Ca^2+^]_cyt_ increase in the presence of 10 µM TCN-201, a NR2A specific inhibitor, revealed that contribution of NR2B to Ca^2+^ accumulation was more prominent in Aβ-treated than in control cells, 4.7 ± 0,4 and 2.6 ± 0.4, quantified as area under the curve, respectively (Fig. 3F; *n* = 3 cultures, 178 cells). To selectively activate synaptic and extrasynaptic NMDARs, primary cortical neurons were treated with bicuculline and 4-AP to activate synaptic NMDARs, followed by MK801-induced blocking. NMDA agonist selectively activated extrasynaptic NMDARs as shown in Fig. 3G (*n* = 3 cultures; 130 cells). Under these conditions, Aβ-treated neurons (*n* = 67 cells) showed specifically a significant increase in synaptic (11.5 ± 1.4 vs. 6.8 ± 0.4 a.u.) but not in extrasynaptic (7.2 ± 0.5 vs. 5.3 ± 0.3 a.u.) NMDA-evoked [Ca^2+^]_cyt_ increase compared to control (*n* = 63 cells) (Fig. 3H). Overall, these results point out that soluble Aβ modify NMDARs-induced cytosolic Ca^2+^ signals by modulating the function of synaptic but not extrasynaptic NR2B-containing NMDARs on neuronal cell surface.

### Aβ oligomers increase both synaptic expression and interaction of NMDA receptor subunit NR2B and postsynaptic scaffold protein PSD-95

**Fig. 4:**
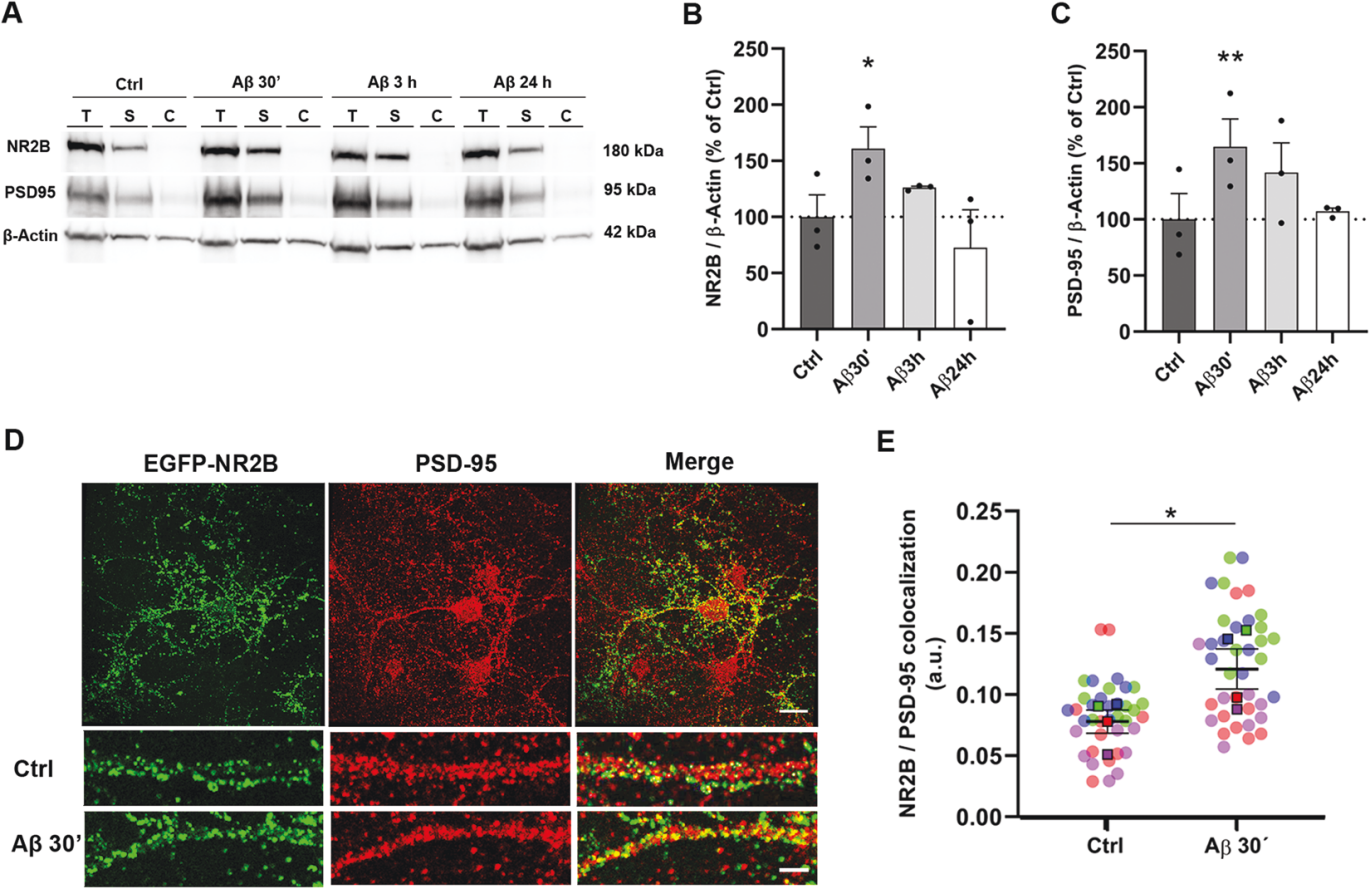
Aβ treatment increases the location of NR2B subunit and PSD-95 in neuronal surface and favors their colocalization. **A** Neurons were exposed to 1 µM Aβ for 30 min, 3 h and 24 h. Total, synaptic and cytosolic protein samples were extracted and NR2B and PSD-95 levels were detected by immunoblot (Supplemental Material). **B, C** Graph bars show the means ± S.E.M of NR2B and PSD95 band volumes normalized to corresponding β-Actin of three independent *p < 0.05, **p < 0.01, one-way ANOVA followed by Dunnett´s test. **D** Neurons, expressing pEGFP-NR2B protein, were labeled in vivo using an antibody against EGFP (green), fixed in 4% PFA and stained with anti PSD-95 in red. Representative photographs of each staining and high magnification photographs of control and Aβ-treatment are shown. **E** Dot plot of Pearson correlation coefficient shows co-assembling of EGFP-NR2B with PSD-95 in dendrites is higher in Aβ-treated neurons. Data are represented as means ± S.E.M. of 52 ROIs from 4 independent experiments. Color dots represent replicates of each experiment. Data were analyzed with paired Student’s *t* test *p < 0.05.

Next, we examined whether Aβ could modify NR2B incorporation at synaptosomal fractions. NR2B subunit levels were significantly increased in Aβ-treated cells for 30 min (Fig. 4A, B, 161 ± 19 % compared to control 100 ± 20%), while no significant changes were observed in Aβ-treated cells either for 3 or 24 h (Fig. 4A,B). Moreover, PSD-95 protein was also overexpressed in Aβ-treated neurons for 30 min (Fig. 4C, 164 ± 25 % compared to control 100 ± 23 %). To explore Aβ-induced modification on NR2B subunit and PSD-95 colocalization in dendrites, EGFP-NR2B subunit was overexpressed in cortical neurons and, after Aβ treatment for 30 min, extracellular region of NR2B subunit was labeled in in vivo conditions using anti EGFP antibody and subsequently stained with the postsynaptic marker PSD-95 (Fig. 4D). Pearson’s coefficient analysis showed that Aβ oligomers increased significantly NR2B/PSD95 colocalization compared to control cells (Fig. 4E, 0.08 ± 0.01compared to 0.12 ± 0.01 a.u., *n* = 4 cultures). Taking together, these results show that NR2B subunit and PSD95 synaptic expression and colocalization are early modulated by Aβ oligomers.

### PKC activation by Aβ oligomers controls NR2B subunit localization and function in neuronal plasma membrane

**Fig. 5:**
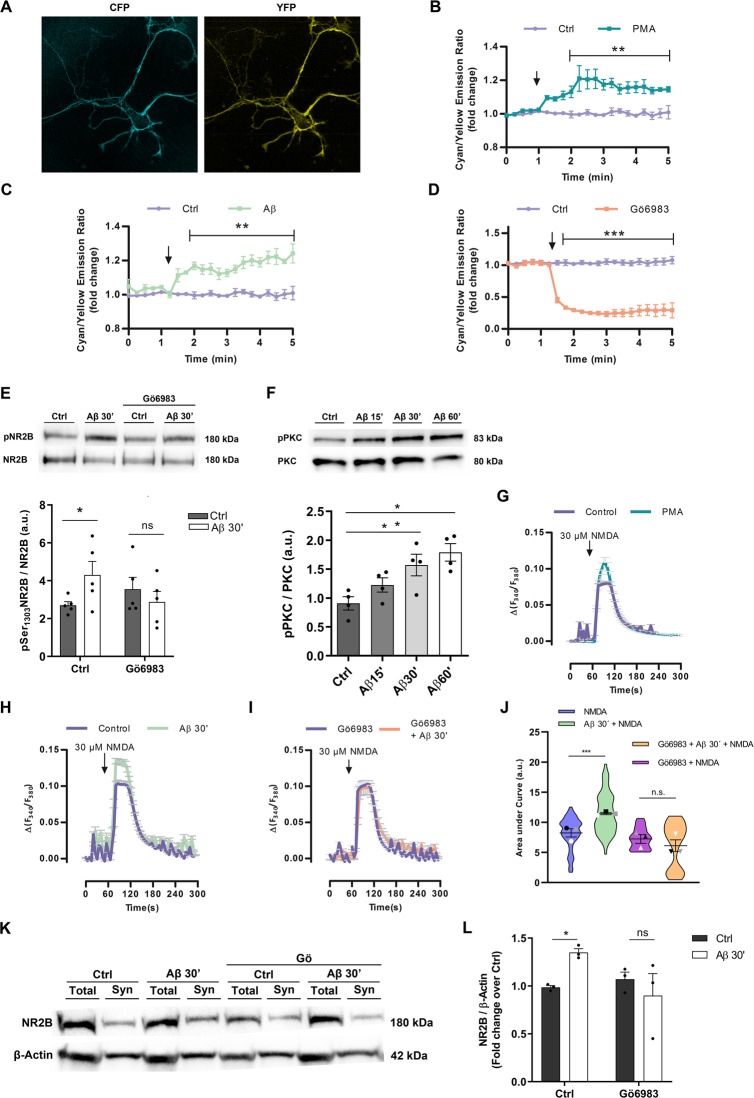
Amyloid β promotes phosphorylation and activation of PKC which controls NR2B surface localization. **A** Images show effective targeting of Myr-Palm CKAR into neuron plasma membranes. **B–D** FRET recordings of PKC activity using Myr-Palm CKAR in neurons after application of 1 mM PMA, 500 nM Gö6983 or 1 µM Aβ plus 100 nM calyculin. Recordings are represented as means ± S.E.M. of three to five experiments. Data were analyzed with two-way ANOVA followed y Sidak´s post hoc test; **p < 0.01, ***p < 0.001. **E** Neurons were pretreated or not with 100 nM Gö6983 for 1 h and stimulated or not with 1 µM Aβ for 30 minutes. Immunoblots show pNR2B at Ser^1303^ in total cell extracts (Supplemental Material). Histogram represents pNR2B normalized with total NR2B. Data were analyzed with two-way ANOVA followed by Bonferroni test *p < 0.05. **F** Neurons were exposed to 1 µM Aβ for 15, 30 and 60 min, and pPKC was examined by western blot (*n* = 5) (Supplemental Material). Histogram represents means ± S.E.M. of band volume intensities of pPKC normalized to total PKC levels. Data were analyzed with with one-way ANOVA *p < 0.05. **G–I** NMDA-mediated Ca^2+^ responses (30 µM, 1 min) in isolated neurons pretreated for 30 min with 1 mM PMA (**G**), with 1 µM Aβ (H) or Aβ together with 100 nM Gö6983 (**I**). **J** Violin plot represents the data distribution and mean ± S.E.M. of area under Ca^2+^ curve for each condition expressed as arbitrary units (a.u.). Data were analyzed with one-way ANOVA followed by Tuckey´s post hot test; ***p < 0.001; *n* = 3 cultures, 262 cells. **K** Neurons were treated with 1 µM Aβ for 30 min in the presence or in the absence of 100 nM Gö6983 and NR2B subunit levels were examined by western blot in total and synaptic fractions (Supplemental Material). **L** Histogram shows quantification of synaptic NR2B in immunoblots (*n* = 3). Data are represented as means ± S.E.M. of band volume intensities normalized to β-actin. Data were analyzed with two-way ANOVA followed by Bonferroni test *p < 0.05.

NMDAR function is regulated by PKC activity [24]. To assess the role of PKC in Aβ-induced NMDAR modulation in neurons, a genetically encoded fluorescent reporter for PKC-mediated phosphorylation (Myr-Palm CKAR, Violin et al. 2003) was transfected in primary neurons. First, basal PKC-mediated FRET signals were confirmed with PMA and Gö6983, PKC activator and inhibitor respectively (Fig. 5A, B, D). Next, PKC activation by 1 μM Aβ oligomers was revealed by FRET signal in CKAR-transfected neurons pretreated with the phosphatase inhibitor calyculin A (100 nM) for 10 min (Fig. 5C) to avoid that cellular phosphatases counteract PKC activity [25]. To confirm whether Aβ oligomers phosphorylate NMDARs by changes in PKC catalytic function by autophosphorylation [26], phosphoPKC and phosphoNR2B (Ser^1303^) were quantified by western blot. Accordingly, we found that 1 μM Aβ oligomers for 30 min significantly increased levels of phosphoPKC in total cell extracts from neurons (Fig. 5F, 1.6 ± 0.2, compared to control levels, 0.9 ± 0.1 a.u; normalized to total PKC) and promoted NR2B phosphorylation at S^1303^ (Figs. 5E, 4.3 ± 0.7 compared to control levels, 2.7 ± 0.2 a.u; normalized to total NR2B). In addition, Gö6983 abolished the Aβ-induced phosphorylation of NR2B subunit in neurons (Fig. 5E). Next, the role of PKC activity in NMDAR function was confirmed by an enhanced intracellular Ca^2+^ influx to NMDA agonist in PMA pretreated neurons (Fig. 5G). Similarly, intracellular Ca^2+^ increase after NMDA addition in Aβ-pretreated neurons was significantly boosted when compared to control (Fig. 5H, J; 11.5 ± 0.4 Aβ-treated compared to control levels 8.5 ± 0.4) and abolished in the presence of the PKC inhibitor Gö6983 (Fig. 5I, J; 8 ± 0.9 Aβ + Gö6983 compared to Gö6983 7 ± 0.4). Finally, we analyzed whether PKC was involved in the Aβ-induced increase of NR2B subunit at synaptic sites. Neurons were incubated with 1 μM Aβ for 30 min, with or without Gö6983, and synaptosomal fractions were analysed by Western blot. As shown in Fig. 5K, L, Aβ oligomers induced a NR2B subunit upregulation with respect to control from 1.0 ± 0.02 to 1.3 ± 0.04, respectively, which was blocked by Gö6983 to 0.9 ± 0.2 compared to its respective control, 1.0 ± 0.1. Altogether, these results indicated that Aβ oligomers control NR2B protein levels in synaptic sites through classic PKCs.

### Aβ oligomers upregulate NR2B subunit density via integrin β1/PKC signalling pathway

**Fig. 6:**
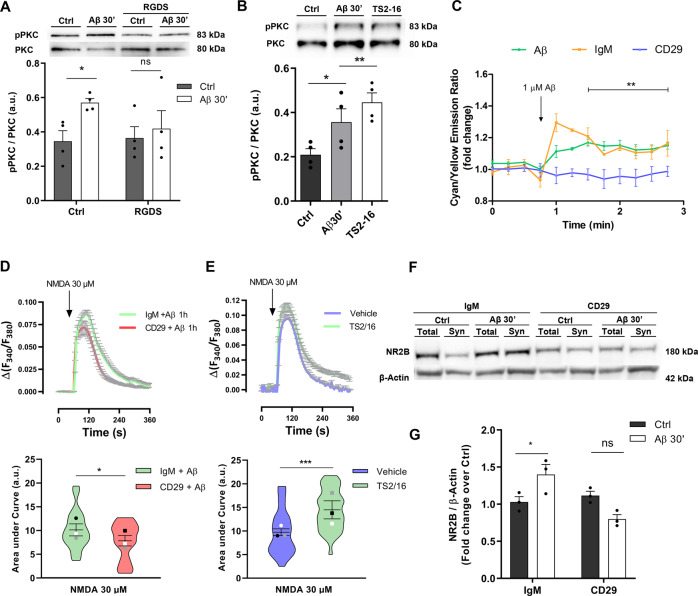
Integrin β1 mediates Aβ-induced PKC activation and surface NR2B expression in primary cortical neurons. **A** PKC phosphorylation was measured by western blot in total cell extracts from neurons previously preincubated with 100 µM RGDS and stimulated with 1 µM Aβ (Supplemental Material). Histogram represents quantification of phosphorylated PKC after normalization with total PKC (*n* = 4). Data were analyzed with two-way ANOVA followed by Sidak´s multiple comparison test; *p < 0.05. **B** Total cell extracts were obtained after treatment with 1 µM Aβ or 0.5 µg/ml TS2-16 for 30 minutes and PKC phosphorylation was analysed by western blot (Supplemental Material). Histogram represents quantification of phosphorylated protein after normalization with total PKC (*n* = 4). Data were analyzed with one-way ANOVA *p < 0.05,i **p < 0.01. **C** Neurons expressing Myr-Palm-CKAR reporter were pretreated with 100 nM calyculin, 0.5 µg/ml isotype control IgM or CD29 antibody, an integrin β1 inhibitor, and Aβ-induced PKC activity was measured by FRET. Recordings are represented as means ± S.E.M. of three to five experiments. Data were analyzed with two-way ANOVA followed y Sidak´s post hoc test; ***p < 0.001. **D, E** Neurons, loaded with Fura-2AM, were preincubated with 0.5 µg/ml IgM or CD29 and exposed to 1 µM Aβ **(D)** or incubated with TS2-16 **(E)** and intracellular Ca^2+^ levels after 30 µM NMDA, 1 min, application were measured by microfluorimetry. Violin plots show data distribution and mean ± S.E.M. of area under Ca^2+^ curve for each condition expressed as arbitrary units (a.u.) of 70-90 cells from at least 3 experiments. Data were analyzed with paired Student’s *t* test *p < 0.05. **F, G** Immunoblot of NR2B subunit levels in functional synaptosomes of primary cortical neurons pretreated with 0.5 µg/ml IgM or CD29 antibodies and stimulated with 1 µM Aβ (n = 4) (Supplemental Material). Data are represented as means ± S.E.M. of band volume intensities normalized to corresponding β-Actin. Data were analyzed with two-way ANOVA followed by Bonferroni test *p < 0.05.

To investigate molecular mechanisms underlying the Aβ/PKC-mediated NR2B subunit modulation, we examined the possible involvement of integrin β1, previously described as a receptor for Aβ oligomers that induce synaptotoxicity and astrogliosis [18, 27]. First, the Aβ-induced PKC phophorylation (Fig. 6A; 0.57 ± 0.02 *vs* 0.35 ± 0.06 a.u., Aβ-treated vs. control cells; normalized to total PKC) was significantly reduced in neurons preincubated with RGDS peptide, which contains the integrin-binding sequence and inhibits its function [28] (Fig. 6B; 0.4 2 ± 0.1 vs. 0.36 ± 0.06 a.u., RGDS + Aβ-treated *vs* RGDS control cells; normalized to total PKC). Similarly, TS2-16, an integrin β1-activating antibody [29] potentiated phosphorylation of PKC to levels comparable to those induced by Aβ oligomers (Fig. 6; 0.44 ± 0.04, 0.35 ± 0.06 vs. 0.21 ± 0.03 a.u., TS2-treated, Aβ-treated *vs* control cells, respectively; normalized to total PKC). Overall, these results indicated that PKC is phosphorylated by Aβ/integrin β1-dependent mechanisms. Moreover, we confirmed the role of integrin β1 in the Aβ-mediated PKC activity by changes in FRET assays with CKAR reporter (Fig. 6C). Thus, PKC activity was blocked when cells were preincubated with antiCD29, an integrin β1-inhibiting antibody [30], but not by the isotype control IgM. Taken together, these results confirmed that Aβ oligomers required integrin β1 to modulate PKC phosphorylation and activation. We also studied whether changes on Ca^2+^ permeability of NMDARs by Aβ oligomers was mediated by integrin β1 (Fig. 6D, E). NMDAR-mediated intracellular Ca^2+^ changes, quantified as area under the curve, in Aβ + IgM antibody-treated cells as control were significantly reduced in neurons exposed to Aβ along with CD29 antibody (Figs. 6D, 10.1 ± 0.9 vs. 7.5 ± 0.8, respectively). Additionally, TS2-16 antibody-treated cells showed a higher Ca^2+^ response to NMDA agonist than vehicle-treated cells (Fig. 6E; 14.2 ± 0.7 vs. 9.7 ± 0.8, respectively). These results confirmed that integrin β1 modulated Ca^2+^ permeability of NMDARs. Finally, we analysed NR2B density in functional synaptosomes of IgM+Aβ- and CD29 + Aβ-treated neurons. Aβ induced an increase in NR2B density in IgM-treated cells (Fig. 6F, G; 1.03 ± 0.07 to 1.4 ± 0.13, IgM control vs. IgM+Aβ) Importantly, the presence of the integrin β1 receptor blocker CD29 antibody inhibited the Aβ-mediated NR2B increase (Fig. 6F,G; 1.12 ± 0.06 to 0.8 ± 0.06, CD29 control vs. CD29 + Aβ). These results suggest that soluble Aβ requires integrin β1/PKC pathway in order to mediate actively on NR2B localization and function in synaptosomes.

## Discussion

Excessive NMDAR activity causes excitotoxicity and promotes cell death, a potential mechanism underlying neurodegeneration in AD [31]. Molecular mechanisms that control the location and function of the NMDAR channels in the progression of AD are still under study. In the prefrontal cortex, senile plaques and neurofibrillary changes start to appear around Braak stage II. However, some studies have demonstrated that the appearance of Alzheimer’s disease neuropathology in this area is preceded by changes in gene expression that point to increased synaptic activity and plasticity [32]. Here, we observed an aberrant increased level of NR2B, PSD95, and synaptophysin in isolated synaptic terminals of AD prefrontal cortex samples at Braak II stages and of the hippocampus of 6-month-age 3xTg-AD mice. These results reveal an early deregulation of synaptic NR2B subunit and PSD95 localization, which consequently would be associated with functional changes on synaptic transmission and/or excitotoxicity. Importantly, low range of Aβ_42_ levels associated with PSD fractions in control, Braak II and III brains positively correlated with synaptic protein expression in human samples. Although there are some fundamental discrepancies between human AD and mouse models, similar results were found in mouse brains. Accordingly, we observed increased expression of NR2B and PSD95 in Aβ-injected mouse brains. Mechanistically, we uncovered that Aβ oligomers required the adhesion protein integrin β1 to promote synaptic location and function of NR2B-containing NMDARs and postsynaptic PSD95 protein through classic PKCs. These changes are induced by brief exposure to soluble Aβ oligomers since longer treatments reduced the NR2B location on the neuronal plasma membrane surface. Further evidence will be needed to identify whether these findings are compensatory mechanisms observed in the early stages of AD [33] or aberrant expression and function of synaptic NMDARs to initiate hyperactivation or excitotoxicity [34].

### Early changes in synaptic terminals in AD brains

Since studies in post-mortem tissues have inherent limitations including looking at a snapshot of the final stage of the disease, analysis of samples with increasing AD neuropathology allows some novel insight into changes that may be occurring in synapses earlier in the degenerative process. Levels of proteins involved in synaptic function decrease in late stages of AD brains including glutamate signaling [35]. Nevertheless, there is little evidence about early changes of NMDAR subunits, PSD-95 and synaptophysin expression in AD. Consistent with data reported here, previous study showed that protein levels of the NR1 and NR2B subunits increase at the early stages of the disease in the hippocampus, mainly in the granular cells of the dentate gyrus, while NR2A levels remain constant [36]. Moreover, in AD brains, PSD-95 varies according to regions, increasing in the frontal cortex, at least in a primary stage, and decreasing in the temporal cortex [37]. Moreover, in brain aging and particularly with AD initiation, Aβ oligomers may bind preferentially to postsynaptic regions, where PSD-95 is present, and even directly to the NMDA receptors, whereby mediate neuronal damage [9, 38, 39]. Aβ oligomers can also trigger neurotoxic pathways via PSD-MAGUK proteins and enhance NMDAR activity by Fyn kinase phosphorylation of NR2B, with the obligate participation of PSD-95 and Tau proteins [40], thereby initiating excitotoxic downstream signaling. Accordingly, we observed that NR2B, PSD95 and synaptophysin levels are higher in PSD and non-PSD fractions specifically in the prefrontal cortex in early AD (Braak stage II) but not in moderate to severe AD. Notably the increased levels of these proteins correlated positively with Aβ_42_ load of the synaptic terminal. These findings raise the intriguing possibility that Aβ may initially cause an increase in synaptic and dendritic markers as a compensatory mechanism for the synaptic deficit, and this phenomenon, though transient, could be the biological basis of the period of mild cognitive impairment seen in AD [41]. Detailed analysis of compensatory/adaptive capabilities of synaptic mechanisms might be a starting point for further discovery new approaches for early diagnostics and treatment of AD.

### Aβ alters the distribution and function synaptic NR2B subunit and PSD-95. Implications in AD

Effects of Aβ on NMDAR subunits at mRNA and protein levels have been extensively studied both in vitro and in vivo. Specifically, Aβ produces a rapid and persistent depression of NMDA-evoked currents and endocytosis of NMDA receptors in cortical neurons through mechanisms involving α−7 nicotinic receptor, protein phosphatase 2B (PP2B) and the striatal-enriched protein tyrosine phosphatase 61 (STEP_61_) [10]. Additionally, STEP_61_ protein levels are progressively increased in the cortex of Tg2576 mice over the first year, as well as in the prefrontal cortex of human AD brains [42], suggesting that NR2B subunit dephosphorylation and endocytosis might be important regulatory mechanisms that lead to neuronal dysfunction and damage during the progression of AD. Here, our data support the novel notion of differential effects of soluble Aβ in synaptic NMDARs during the progression of the pathology. In particular, we found that Aβ oligomers increased the presence and functionality of NR2B- but not NR2A-containing NMDARs in synaptosomes of cultured neurons without modifying the total levels of both subunits. Disruption of this equilibrium has been linked to abnormal brain development and neuropsychiatric disorders such as AD and schizophrenia [43]. Accordingly, NR2 subunits regulate pro-survival and pro-death signaling [44], synaptogenesis, synaptic pruning, and synapse stabilization in the CNS [45]. Specifically, it has been proposed a model in which NR2A acts as a stabilizing force in the synapse, making both functional and structural changes more difficult, whereas NR2B controls the structural changes, such as new spine formation and spine retraction [45]. Moreover, a change in the ratio of NR2A to NR2B affects subsequent NMDAR-dependent synaptic modifications. Manipulations of the NR2A-to-NR2B ratio regulate both the magnitude and sign of subsequent plasticity, leading to a shift in the LTP and LTD. In agreement with a critical role of NR2B in LTP, when the ratio of NR2A to NR2B increases, stronger stimulation is required to induce LTP, whereas a wider range of weaker stimulations can induce LTD [46].

PSD95 promotes the insertion of new NMDARs, stabilizes receptors in the plasma membrane, and enhances channel opening rate [47]. In parallel to the NR2B subunit increase, PSD-95 density was also increased in isolated synaptic terminals after a short Aβ treatment in neurons. However, previous reports showed that Aβ peptide reduces total and synaptic PSD95 levels by inducing its degradation [48, 49]. Data could be different from those obtained in this work because the conformation of Aβ peptide used for neuronal treatments was mostly monomeric [48] or Aβ concentration was extremely high [49]. This abnormal increase of NR2B-containing NMDARs and PSD-95 on synaptic terminals in the early stages of the AD could initiate functional changes on synaptic plasticity, intracellular Ca^2+^ levels together with important structural changes on spines [27], and subsequently contribute to the aberrant synaptic function.

### Integrin β1 and PKC in AD pathology

PKC increases the number of functional NMDARs at the cell surface of oocytes and neurons and increases NMDAR channel opening rate [50], trafficking, and gating [51]. Our results showed that Aβ oligomers mediate PKC activation in order to modulate both NR2B expression and the rise of NMDA-induced [Ca^2+^]_cyt_. NR2B subunit phosphorylation at S^1303^ enhances the NR1/NR2B receptor channel conductance and contributes to excitotoxic signaling [52]. Here, our data describe that PKC phosphorylates NR2B subunit at S^1303^ residue, suggesting that Aβ could regulate the NMDAR location as well as its activity in a PKC-dependent manner. Furthermore, our results suggest that the increased cell surface location and dysfunction of NR2B-containing NMDARs could be explained by the abnormal increase of the colocalization of PSD-95 and phosphorylated NR2B subunit into the synaptic terminals.

Interaction between integrins and Aβ peptides promote spine and dendritic changes [27], synaptic dysfunction [53], gliosis [18] and oligodendrocyte differentiation [20]. We show that Aβ oligomers activate PKC and induce changes in NR2B phosphorylation, location, and NMDAR function through integrin β1. In conclusion, we have deciphered a new molecular mechanism underlying synaptic dysfunction, which highlights the integrin β1/PKC/NMDAR signaling axis as an early event in AD. We postulate that intermediaries in the signaling cascade described in this study can become promising biomarkers in AD progression and potential pharmacological targets to block the toxic program activated by β-amyloid peptide.

## Material and methods

### Selection of cases and general human sample processing

Frozen samples from the prefrontal cortex were obtained from the Clinic Hospital-IDIBAPS Biobank, following the guidelines of Spanish legislation in this matter and the approval of Ethic Committee of the University of the Basque Country (UPV/EHU). Details of data collection and criteria used to classify the samples are provided in Supplementary Methods. AD samples were grouped by Braak and Braak classification [22] into AD‐II, AD‐III, AD‐IV, and AD‐V‐VI (supplementary Table 1).

### Animals

Experiments were performed in Sprague Dawley rats, C57bl/6 mice and in the triple transgenic mouse model of Alzheimer´s disease (3xTg‐AD), which harbors the Swedish mutation in the human amyloid precursor protein (APPSwe), presenilin knock‐in mutation (PS1M146V), and TauP301L mutant transgene (TauP301L) [23]. All the animals used in this study were chosen randomly. All experimental procedures were conducted under the supervision and approved by the Ethic Committees of the University of the Basque Country (UPV/EHU). Animals were housed in standard conditions with 12 h light cycle and with *ad libitum* access to food and water, in accordance with the European Communities Council Directive. All possible efforts were made to minimize animal suffering and the number of animals used.

### Primary cortical neuron culture, transfection, and immunofluorescence

Cortical neurons were isolated from the cortical lobes of E18 Sprague-Dawley rat embryos according to previously described procedures (Supplemental material; [54]). To analyze NR2B localization, 2 × 10^6^ cortical neurons were transfected with 3 µg pEGFP (Clontech Laboratories, Inc, CA, USA) or 3 µg pEGFP-NR2B using Rat Neuron Nucleofector Kit (Lonza, Switzerland) according to the manufacturer’s instructions and seeded and maintained as described above. pCI-EGFP-NR2b wt was a gift from Andres Barria & Robert Malinow (Addgene plasmid #45447; http://n2t.net/addgene:45447; RRID:Addgene_45447), [55]. At 9 DIV, living neurons were incubated with chicken anti GFP (Aves Labs, Inc, #GFP1020) in culture medium at 37 °C for 20 min and fixed in 4% paraformaldehyde (w/v) in PBS for 5 min. Cells were incubated for 30 min in blocking solution (PBS pH 7.5, 4% NGS, 0.05% Triton X-100) and rabbit anti-PSD95 (1:500, abcam, #ab18258) was used. Secondary antibodies coupled to Alexa 488 or Alexa 594 (1:500), were purchased from Invitrogen (Thermo Fisher Scientific, goat anti-chicken Alexa Fluor-488 #A11039 and goat antirabbit Alexa Fluor-594 #A11005) [56].

### Mouse intrahippocampal injections, immunofluorescence and image analysis

Adult male mice (3–4 months) were randomized, anesthetized with ketamine hydrochloride (80 mg kg^−1^) and xylazine (10 mg kg^−1^), were injected stereotaxically into the hippocampus at the following coordinates: 2.2 mm from Bregma, 1.5 mm lateral to the sagittal suture, and 2 mm from the pial surface. Mice were divided into two groups (*n* = 6 per group) and injected with 3 µl of vehicle (DMSO 17%, Ham’s F-12 83%) or Aβ oligomers (135 ng). After 7 days, mice were fixed and processed as described previously [18] (Supplemental M&M).

### Neuronal protein extract preparation

For total neuron protein preparation, 6 × 10^5^ cells were washed twice in cold 0.1 M PBS, scraped in sample loading buffer (62.5 mM Tris pH 6.8, 10% glycerol, 2% SDS, 0.002% bromophenol blue, and 5.7% β-mercaptoethanol in dH_2_O) and boiled at 95 °C for 5 min.

For isolating synaptosomes, neurons 1 × 10^6^ were washed in cold 0.1 M PBS twice, scraped in 200 μl of Syn-PER^TM^ Synaptic Protein Extraction Reagent with Halt^TM^ protease inhibitors cocktail (Thermo Fisher Scientific, MA, USA) and centrifuged at 1200 × *g* for 10 minutes at 4 °C. Pellet was resuspended in 40 μl of synaptosome buffer and the supernatant was centrifugated at 15,000 × *g* for 20 minutes at 4 °C. The supernatant was saved as the cytosolic fraction and the last pellet was resuspended in 40 μl of synaptosome buffer and saved as synaptosomal fraction.

### Biotinylation of neuronal surface proteins

Biotinylation assays were performed according to the methodology described previously [57]. Briefly, cortical neurons (9 DIV), in 100 mm diameter petri dishes, were cooled on ice and washed twice in cold 0.1 M PBS pH 7.5 containing 0.5 mM MgCl_2_ and 1 mM CaCl_2_ (Mg^2+^/Ca^2+^ PBS). For membrane protein biotinylation, neurons were incubated in PBS containing 1 mg/ml EZ-Link Sulfo^TM^-NHS-LC-biotin (Thermo Fisher Scientific, MA USA) for 20 min at 4 °C with continuous gentle agitation. Afterwards, cells were rinsed three times in cold Mg^2+^/Ca^2+^ PBS supplemented with 50 mM glycine and 0.5% bovine serum albumin (BSA), washed twice with cold Mg^2+^/Ca^2+^ PBS and lysed in RIPA buffer (50 mM TrisHCl pH 7.5, 150 mM NaCl, 0.5% Sodium deoxycholate, 0.1% SDS, 1% Nonidet P-40) with Halt^TM^ protease inhibitors cocktail (Thermo Fisher Scientific, MA,USA). Ten percent of the lysate was used for protein quantification, while remainder volume was solubilized with rotation on a wheel at 4 °C for 2 hr. Next, cell and nuclear debris were removed by centrifugation at 14,000× *g* for 15 min. The supernatant was incubated with Pierce^TM^ NeutrAvidin^TM^ agarose (Thermo Fisher Scientific, MA, USA) using a ratio 3:1 of cell lysate and avidin beads to purify biotinylated proteins. The suspension was then washed twice with RIPA buffer supplemented with 500 mM NaCl followed by a further wash in standard RIPA buffer. Biotinylated proteins were eluted with sample loading buffer by heating at 95 °C for 5 min.

### Subcellular fractionation of brain tissues

Subcellular fractionation was performed following a previously described method [58]. Brain tissues from both animal and human samples were homogenized in ice sucrose buffer (10 mM HEPES pH 7.4, 0.32 M sucrose), and centrifuged at 1000× *g* for 10 min at 4 °C. Supernatant (total protein fraction) was centrifuged at 14,000× *g* for 20 min at 4 °C to obtain crude synaptosomal fraction and cytosolic protein enriched supernatant. The pellet was washed twice with washing buffer (4 mM HEPES, 1 mM EDTA, pH 7.4) by resuspension and centrifugation at 12,000× *g* for 20 min at 4 °C, and then resuspended in buffer A (20 mM HEPES pH 7.2, 100 mM NaCl, 0.5% Triton X-100). After rotation for 1 h at 4 °C, suspension was centrifuged at 12,000× *g* for 20 min at 4 °C to yield a non-PSD fraction containing extrasynaptic proteins. The resultant pellet was washed twice and resuspended in buffer B (20 mM HEPES pH 7.5, 0.15 mM NaCl, 1% Triton X-100, 1% SDS, 1 mM dithiothreitol, 1% deoxycholate) for 1 h at 4 °C, followed by centrifugation at 10,000× *g* for 20 min at 4 °C to obtain the PSD fraction containing synaptic proteins. All buffers were freshly supplemented with protease inhibitors cocktail (Thermo Fisher Scientific, MA, USA), 100 mM Na_3_VO_4_ and 100 mM phenylmethylsulfonyl fluoride (PMSF) prior to use, and fractions were stored as aliquots at −80 °C. Samples were quantified with a detergent-compatible assay reagent (Pierce BCA Protein Assay Kit) according to the manufacturer’s instructions (Thermo Fisher Scientific, MA, USA).

### Human Aβ_1-42_ ELISA assay

Human Aβ_1-42_ levels were quantified in synaptosomal PSD fractions of human and mouse samples using a sandwich ELISA kit (Invitrogen, #KHB3441) according to the manufacturer’s protocol. Aβ_1-42_ concentration was calculated from the standard curve and normalized to the total protein concentration.

### Western Blotting

Protein lysates from cortical neurons, 3xTg-AD mice and human brains were separated by SDS-PAGE using 7.5% Tris-Glycine polyacrylamide gels (Bio-Rad). Electrophoresis was conducted in a Tris-Glycine buffer (25 mM Tris, pH 8.3, 192 mM glycine, 0.1% SDS in dH_2_O) by using the Criterion cell system (Bio-Rad). Blots were developed with rabbit anti-NR2B (1:1000, Millipore, #AB1557P), rabbit anti-pSer^1303^NR2B (1:1000, Millipore, #07-398), rabbit anti-NR2A (1:1000, Millipore, #AB1555P), rabbit anti-PSD95 (1:500, abcam, #ab18258), mouse anti-synaptophysin (1:500, Millipore, #MAB329), rabbit antiphosho PKC (1:1000, Cell Signaling, #9371), rabbit anti-PKC (1:1000, abcam, #ab179521) and rabbit anti-β-actin (1:5000; Sigma-Aldrich, #A2066). Secondary antibodies conjugated with horseradish peroxidase (HRP) were purchased from Sigma (1:5000, sheep antimouse-HRP #A6782, goat antirabbit-#HRP A6154).

### Measurement of intracellular Ca^2+^ concentration

Neurons were preincubated with 5 µM fura‐2 AM (Invitrogen, Thermo Fisher Scientific) at 5 μM in culture medium for 30 min at 37 °C and washed as previously described [9; Supporting Information].

### Fluorescence Resonance Energy Transfer (FRET) assays

Neurons were transfected with Myr-Palm C kinase activity reporter (MyrPalm-CKAR). MyrPalm-CKAR was a gift from Alexandra Newton (Addgene plasmid # 14862; http://n2t.net/addgene:14862; RRID:Addgene_14862) [25]. At 8 DIV, transfected neurons were transferred to incubation buffer (HBSS containing 20 mM HEPES, pH 7.4, 10 mM glucose, and 2 mM CaCl_2_) and PKC activity was imaged using a TCS SP8X confocal microscope (Leica, Germany). Briefly, cells were excited at 458 nm (argon laser line), and CFP and YFP emission were acquired by HyD detectors for FRET ratio quantification at an acquisition rate of 1 frame/15 s during 5 min. The pinhole was set at 300 μm and images were taken with a 63X oil objective (1.4 NA). For data analysis, a homogeneous population of 3–5 cells was selected in the field and neuronal somata membrane selected as ROI. Background values were always subtracted and data are expressed as R/R_0_ ± S.E.M. (%) in which R represents the CFP/YFP fluorescence ratio for a given time point and R_0_ represents the mean of the resting FRET ratio.

### Statistical analysis

All data were expressed as mean ± S.E.M. Statistical analysis were performed using absolute values. GraphPad Prism software was used applying one-way or two-way analysis of variance (ANOVA) followed by Bonferroni´s, Sidak´s and Tuckey´s post hoc tests for multiple comparisons, paired two-way ANOVA for repeated measures and two-tailed, unpaired Student’s *t* test for comparison of the two experimental groups.

## Supplementary information

Supplemental Information

Check List

Original Western Blots

## Data Availability

The data used in this study are available from the corresponding author upon reasonable request.
