# Supplementary material for: Amyloid β / PKC-dependent alterations in NMDA receptor composition are detected in early stages of Alzheimer´s disease
Source: Cell Death Dis. 2022 Mar 19;13(3):253. doi: 10.1038/s41419-022-04687-y (PMC8934345; doi:10.1038/s41419-022-04687-y)
Supplement: Supplementary file 1 — Supplemental Information [file 41419_2022_4687_MOESM1_ESM.docx]

**Amyloid β / PKC-dependent alterations in NMDA receptor composition are detected in early stages of Alzheimer´s disease**

**Supplementary Methods**

**Preparation of oligomeric forms of Aβ_1‐42_**

Aβ_1-42_ (Bachem, Switzerland) was initially dissolved to 1 mM in hexafluoroisopropanol (Sigma‐Aldrich, Merck, Germany), which was removed in a speed vacuum system and the peptide film was stored dried at ‐80°C. For the aggregation protocol, the peptide was resuspended in anhydrous DMSO (Sigma‐Aldrich, Merck, Germany) to a concentration of 5 mM. Finally, the peptide was diluted at 100 μM in Hams F‐12 (PromoCell GmbH, Germany) and incubated at 4°C for 24h.

**Mouse intrahippocampal injections, immunofluorescence and image analysis**

### Coronal 40 μm‐thick sections were permeabilized and blocked with 0.25% Triton X‐100, 3% NGS in 0.1 M PBS for 1 h at room temperature (RT) and were incubated with either rabbit anti-NR2B (1:200, Synaptic Systems, #244103) or mouse anti-PSD95 (1:100, Synaptic Systems, #124011) antibodies overnight at 4°C with gently shaking. Slices were then washed three times in 0.1 M PBS for 10 min and incubated with blocking solution containing fluorochrome‐conjugated antibodies at RT for 1 h (goat anti-rabbit Alexa Fluor-594, #A11012 and sheep anti-mouse Alexa-Fluor-594 #A11005). After that, slices were washed three times in 0.1 M PB for 10 min, and second wash contained DAPI (4 μg/ml). Finally, slices were mounted on glass slides with Fluoromount‐G mounting medium (SouthernBiotech, Birmingham, AL, USA). Images of dentate gyrus from 2-3 sections per animal were taken with a Leica TCS SP8 laser scanning microscope using 63X oil‐immersion objectives (Leica microsystems, Mannheim, Germany). Mean gray value along three region of interest (ROIs) of 27 x 27 μm per image was quantified with ImageJ-FIJI.

**Primary cortical neuron cultures**

Briefly, cortical tissue was enzymatically digested with 0.25% trypsin and 0.004% deoxyribonuclease in Hank´s balanced salt solution (HBSS), mechanically dissociated by using needles, and filtered through a 40 μm nylon mesh. Neurons were resuspended in B27 Neurobasal medium with 10% FBS and seeded onto poly-L-ornithine-coated plates or glass coverslips at 1.5 x 10^5^ cells per well. For single cell imaging experiments, cells were seeded onto glass-bottom μ-dishes (Ibidi GmbH, Germany). The medium was replaced by serum-free, B27-supplemented Neurobasal medium 24 hours later.

In excitotoxicity experiments, cortical neurons at 8–10 days in culture were pretreated with Aβ 1 µM for 30 min or 24h and exposed to NMDA 30 µM in HBSS (free of Ca^2+^ and Mg^2+^) containing 2.6 mM CaCl2, 10 mM glucose, and 10 µM glycine in supplemented Neurobasal® for 30 min at 37 ºC and washed. In all cases. Cell damage was estimated 24 h later by measuring the level of lactate dehydrogenase released (LDH; Cytotox 96®, Promega, Madison, WI) from damaged cells into the culture media. Data were normalized to the activity of LDH released from vehicle-treated cells and calculated as the fold change of the control. Results were expressed as the means ± SEM of independent experiments performed in triplicates.

**Measurement of intracellular Ca^2+^ concentration**

Experiments were performed in a coverslip chamber, mounted on the stage of a inverted epifluorescence microscope (Zeiss Axiovert 35) equipped with a 150 W xenon lamp Polychrome IV (T.I.L.L. Photonics) and a Plan Neofluar 40X oil‐immersion objective (Zeiss). Cells were visualized with a high resolution digital black/white CCD camera (ORCA C4742‐80‐12 AG; Hamamatsu Photonics Iberica, Spain). Calcium levels were estimated by the 340/380 ratio method and data were analyzed with Excel (Microsoft Corporation, WA, USA) and Prism software (GraphPad Software, Inc, CA, USA).

**Supplementary table and figures**

**
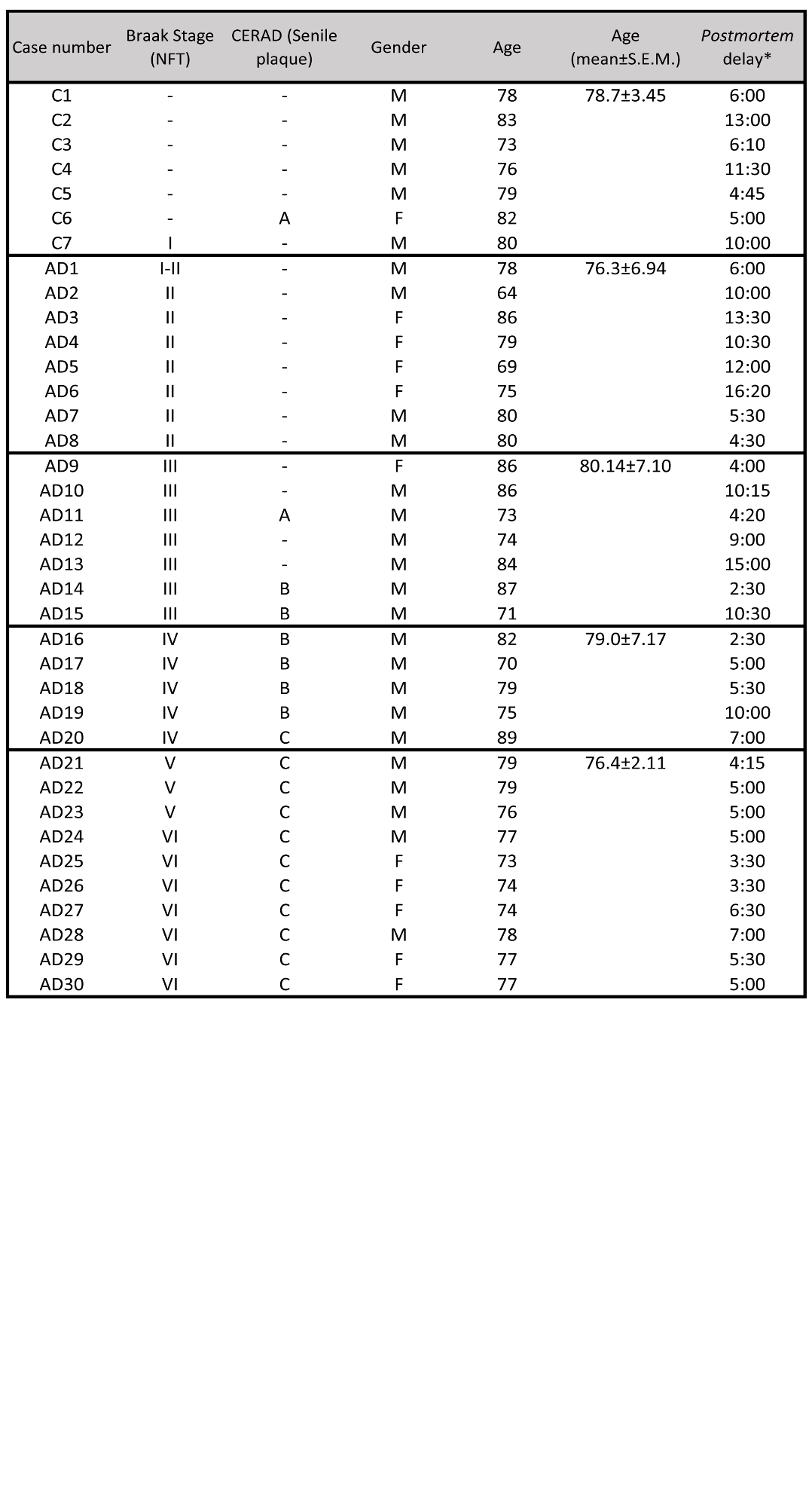
**

**Table S1. Clinical and pathological characteristics of subjects providing post-mortem tissues used for western blot and ELISA study.**

Informed consent was obtained from all subjects. M: male, F: female; Post-mortem delay: hours; C: control, AD: Alzheimer´s disease; NFT: neurofibrillary tangle pathology stages I-VI of Braak; CERAD: β-amyloid deposition in the form of diffuse and/or neuritic plaques Braak stages: no deposits to C.


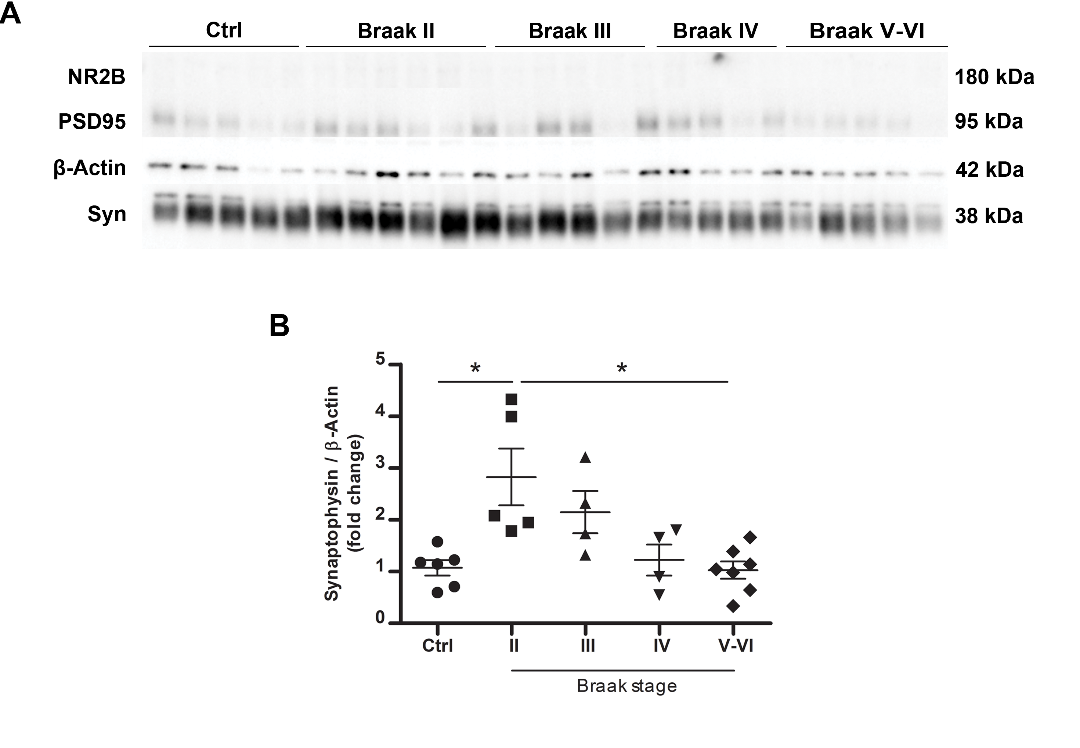


**Figure S1. Synaptophysin levels increase in non-PSD fraction of prefrontal cortex from AD patients at early disease stages (A)** Western blot of NR2B, PSD-95 synaptophysin and β-actin in non-PSD fraction of *post-mortem* prefrontal cortex from controls and AD patients at different Braak stages (Braak II-VI). **(B)** Scatter plot showing synaptophysin in controls (Ctrl) and AD subjects (n = 4-7 per group). Data were analysed with one-way ANOVA followed by Bonferroni´s test; *p<0.05


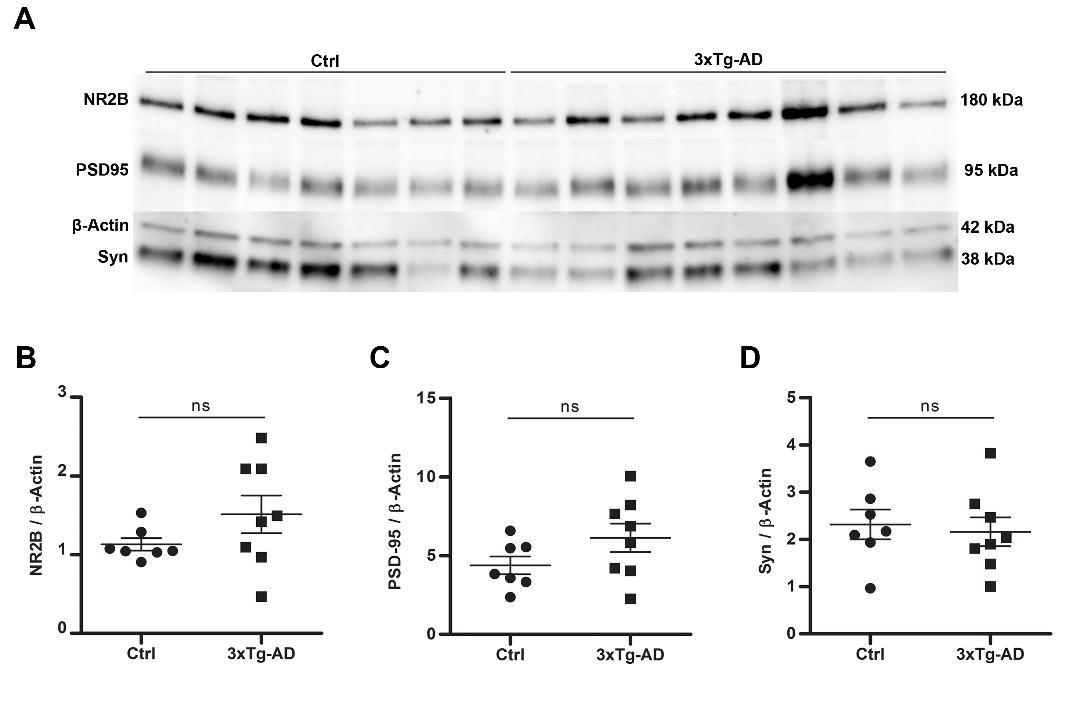


**Figure S2.** **NR2B, PSD95 and synaptophysin levels remain similar in 1-month-old 3xTgAD mouse synaptosomes as compared to controls. (A-D)** Western blots and quantitative analysis of NR2B, PSD-95, and synaptophysin in isolated synaptic terminals of 1-month-old control and 3xTg-AD mice (n=7 animals per group). Scatter plots represented as means ± S.E.M. of volume values normalized to corresponding β-Actin; *p<0.05, **p<0.01; un-paired Student’s t test

**
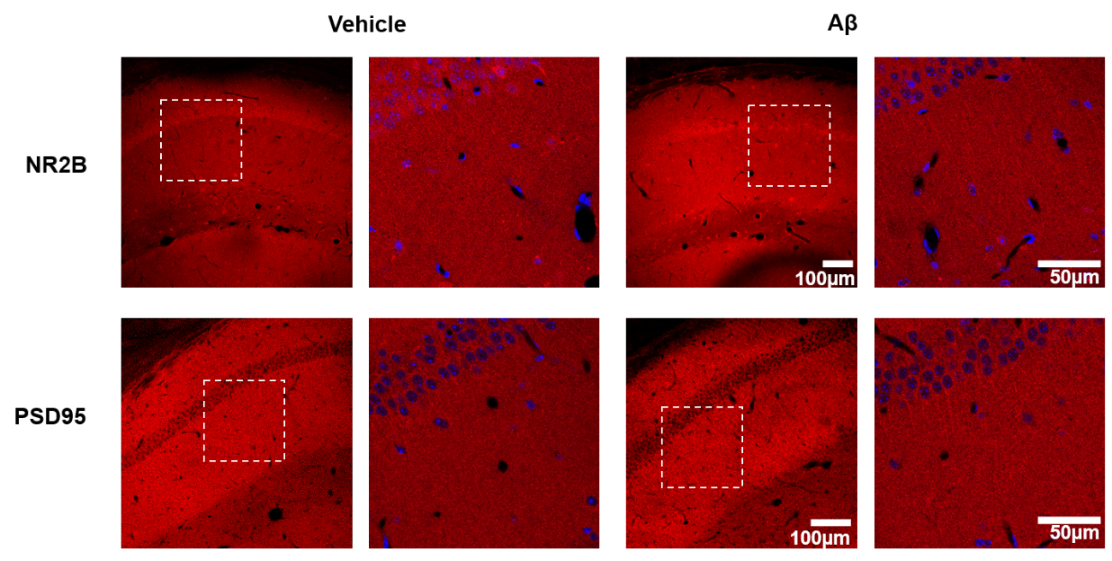
**

**
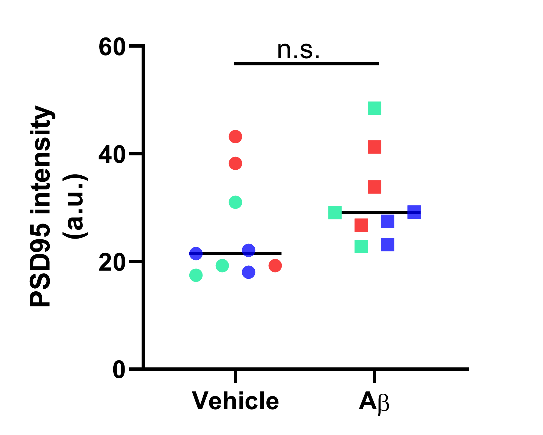

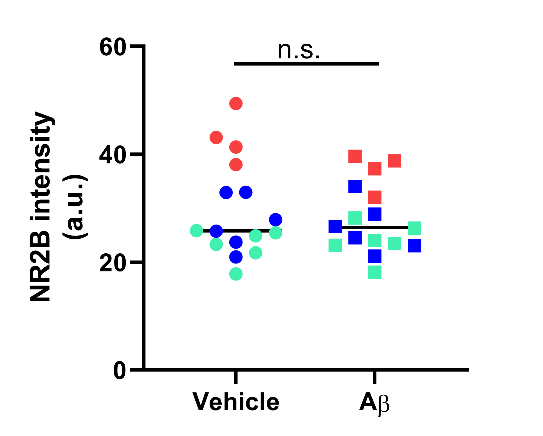
**

**Figure S3**: Coronal sections of mouse brains were analyzed after 7 days of vehicle and Aβ (135 ng) injection. Photomicrographs show NR2B and PSD95 immunolabeling in CA1. Scatter dot plots show the mean values of NR2B and PSD95 intensities in vehicle- and Ab-injected mice. 2-3 brain sections of 9-16 mice were used. Data were analyzed with un-paired Student´s t-test.

**
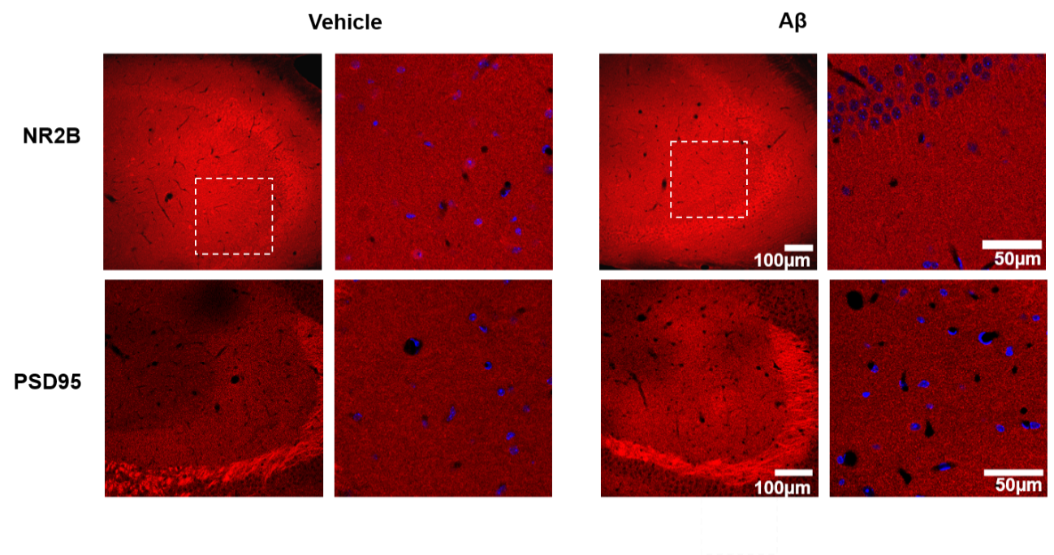
**

**
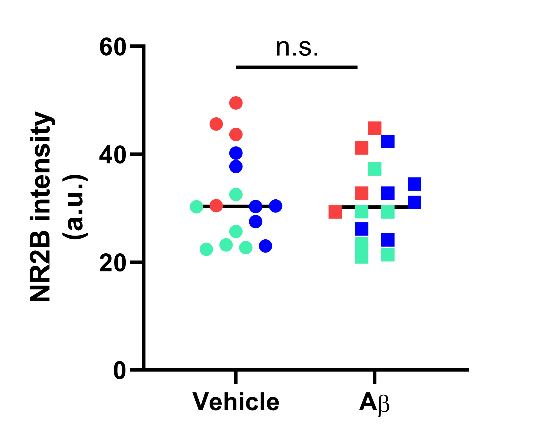

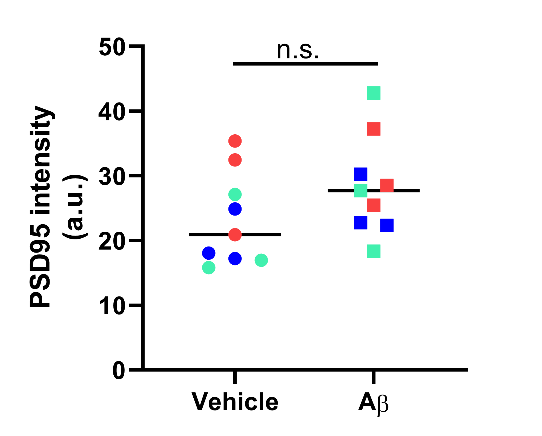
**

**Figure S4**: Coronal sections of mouse brains were analyzed PSD95 immunolabeling in CA3. Scatter dot plots show the mean values of NR2B and PSD95 intensities after 7 days of vehicle and Aβ (135 ng) injection. Photomicrographs show NR2B and in vehicle- and Ab-injected mice. 2-3 brain sections of 9-16 mice were used. Data were analyzed with un-paired Student´s t-test.

**
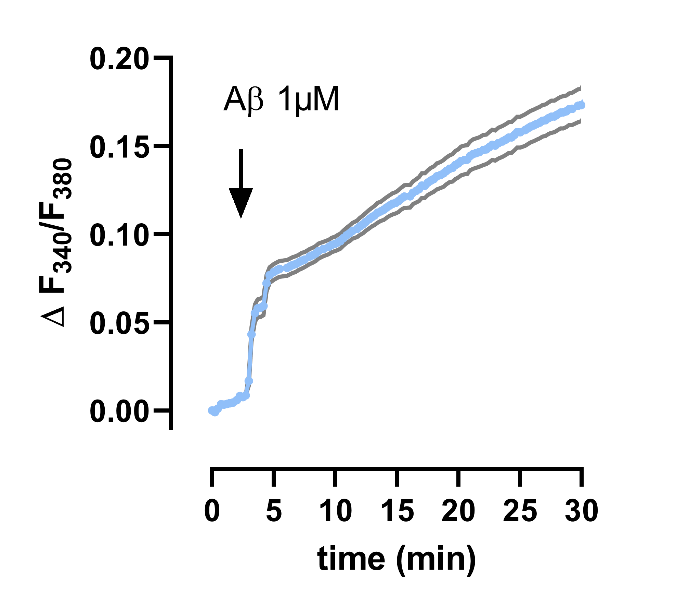

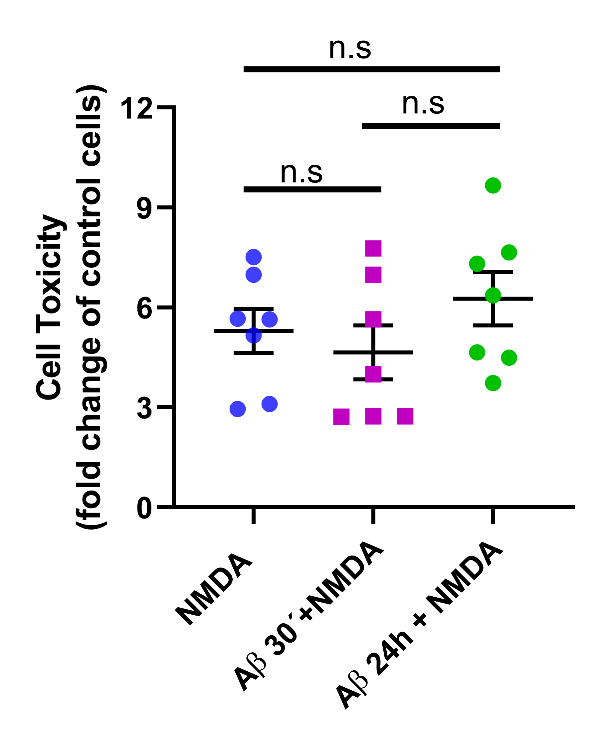
A** **B**

**Figure S5:** (A) Fura-2 loaded neurons were exposed to Ab 1 µM and cytosolic Ca^2+^ load quantified. Trace show normalized means ± SEM of 78 cells of three independent cultures. (B) Toxicity of NMDA (30 min; 30 μM) in cultured cortical neurons as measured 24 h later with the LDH viability assay. Data represent mean ± SEM of the LDH signals in *n* = 7 cultures, expressed as a fold change of control untreated levels.
