# Supplementary material for: Amyloid β / PKC-dependent alterations in NMDA receptor composition are detected in early stages of Alzheimer´s disease
Source: Cell Death Dis. 2022 Mar 19;13(3):253. doi: 10.1038/s41419-022-04687-y (PMC8934345; doi:10.1038/s41419-022-04687-y)

Figure 1A.

αNR2B (˜180 KDa)

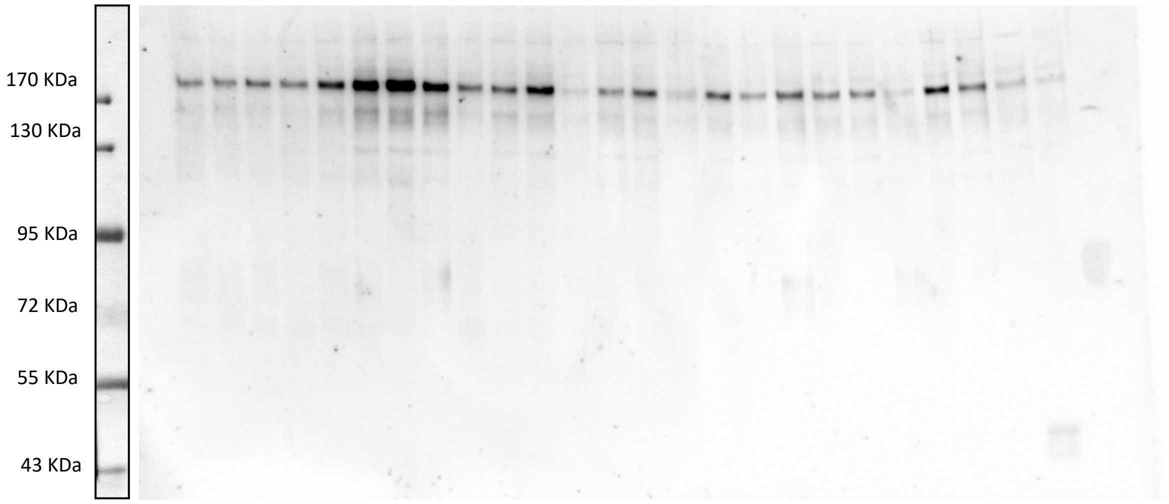

αPSD95 (~ 95KDa)

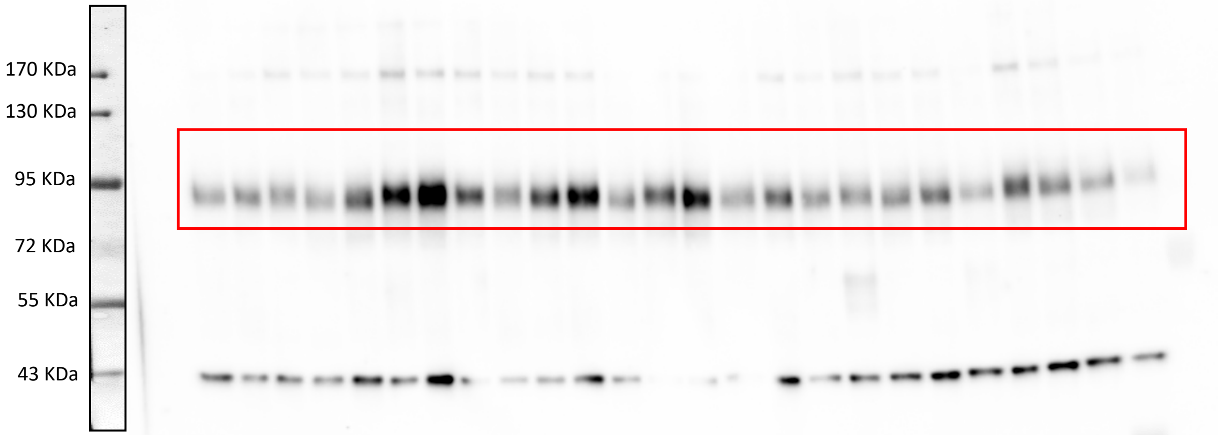

αβ-Actin (~ 42KDa)

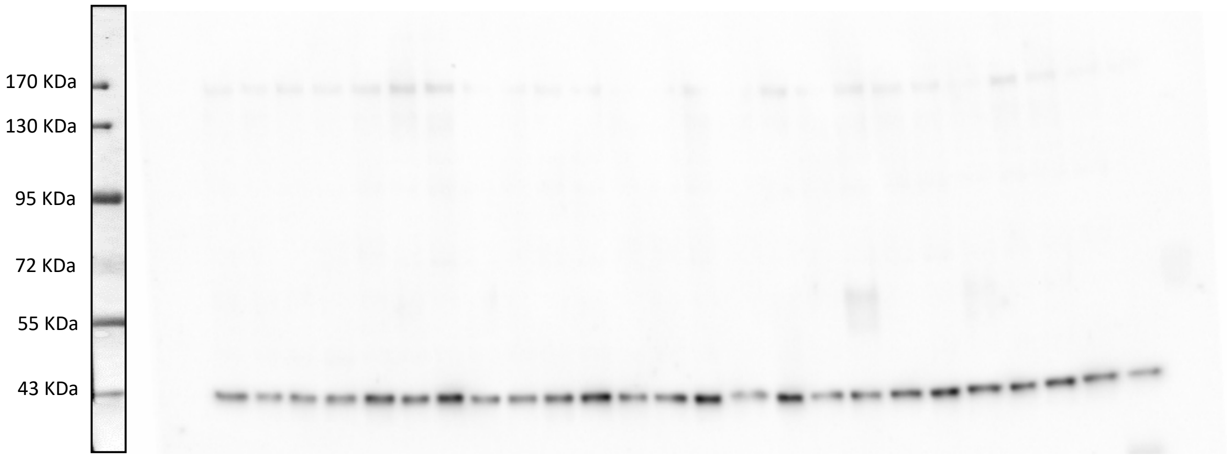

αSyn (~ 38KDa)

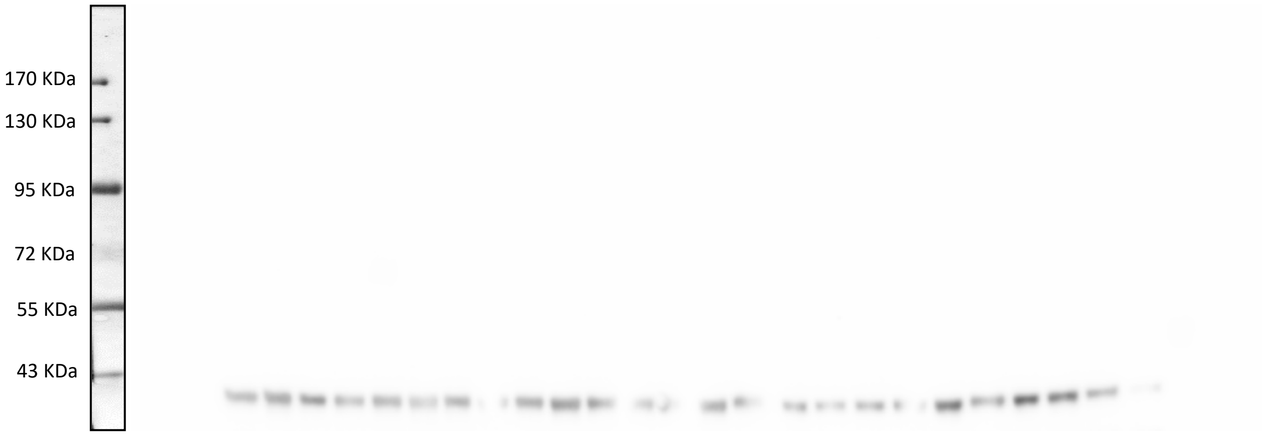

Figure 2A.

αNR2B (~ 180KDa)

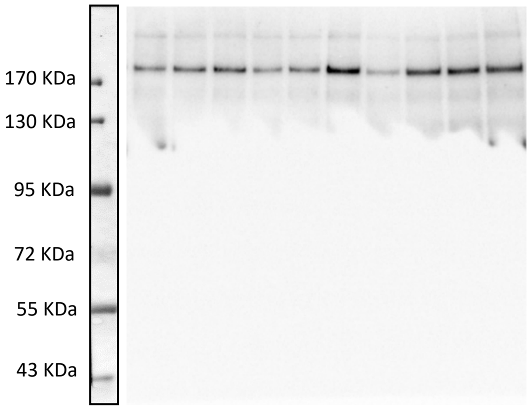

αPSD95 (~ 95KDa)

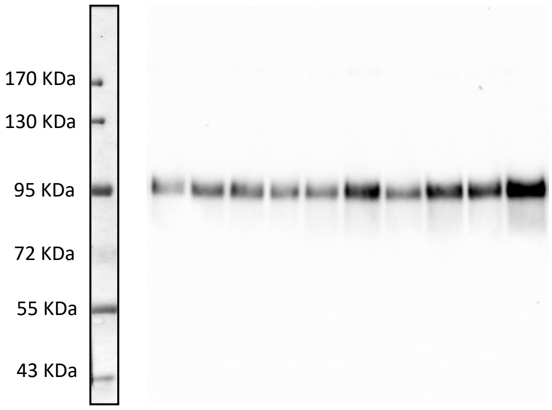

αβ-Actin (~ 42 KDa) and **αSyn** (~ 38 KDa)

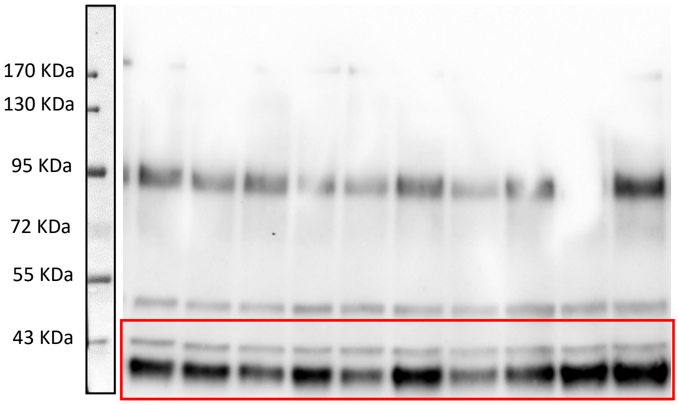

Figure 3A.

αNR2B (~ 180KDa)

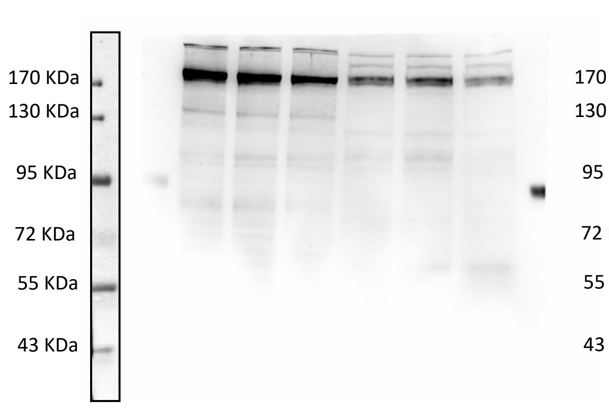

αβ-Actin (~ 42 KDa)

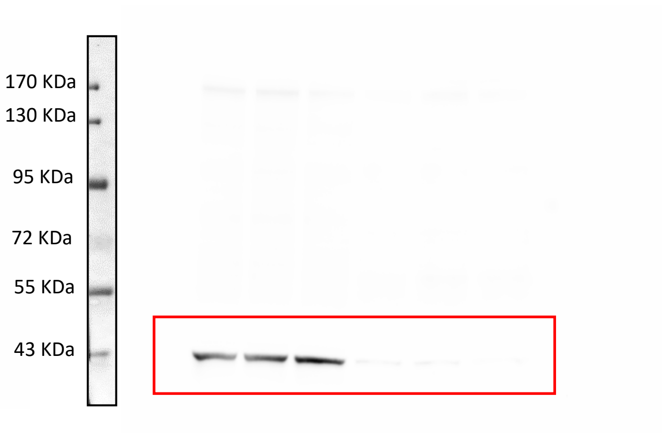

αNR2A (~ 180KDa)

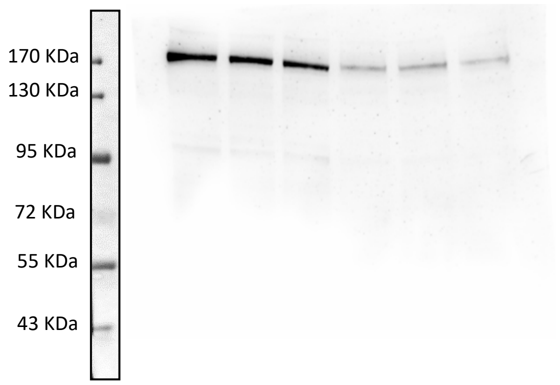

αβ-Actin (~ 42 KDa)

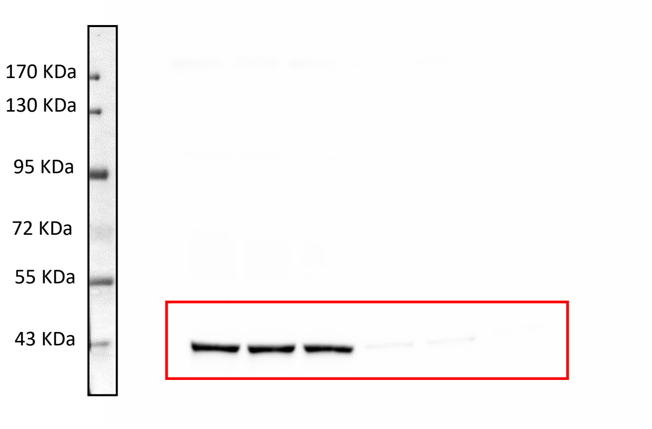

Figure 4A.

αNR2B (~ 180KDa)

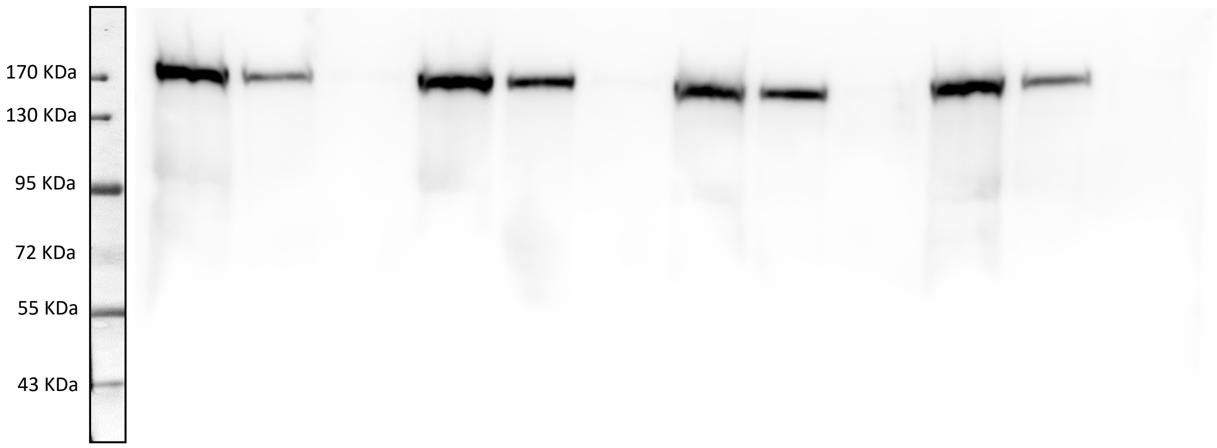

αPSD95 (~ 95KDa)

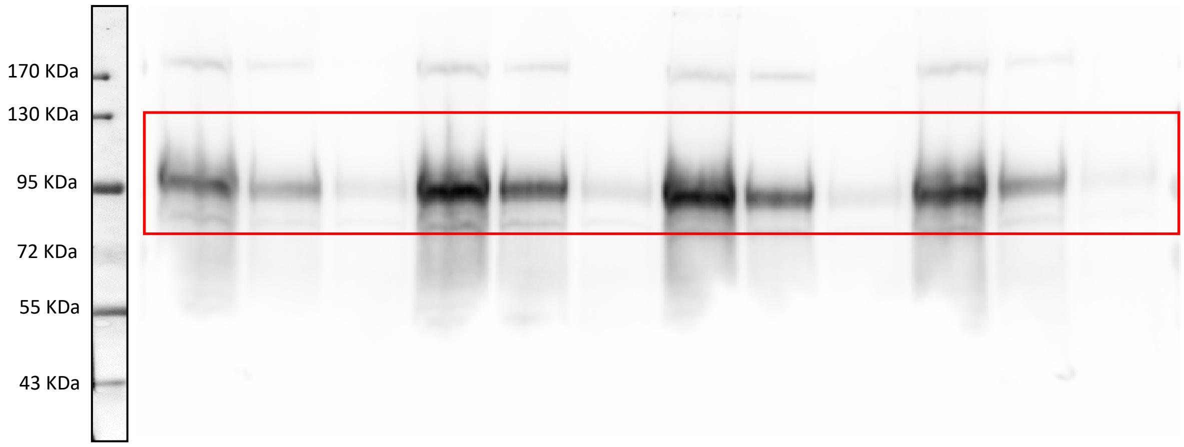

αβ-Actin (~ 42KDa)

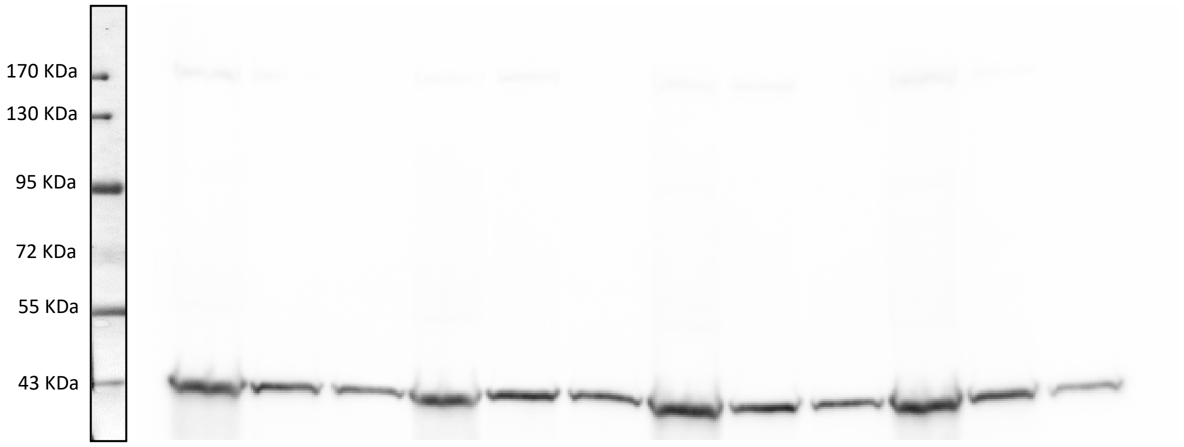

Figure 5E.

αpNR2B (~ 180KDa)

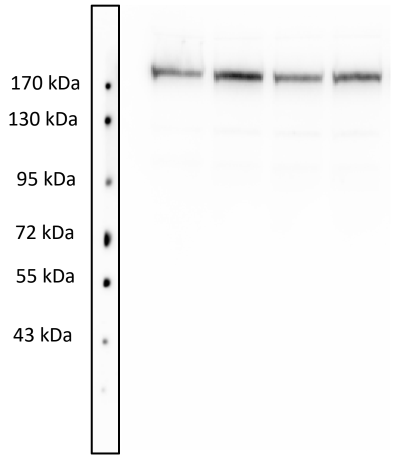

αNR2B (~ 180KDa)

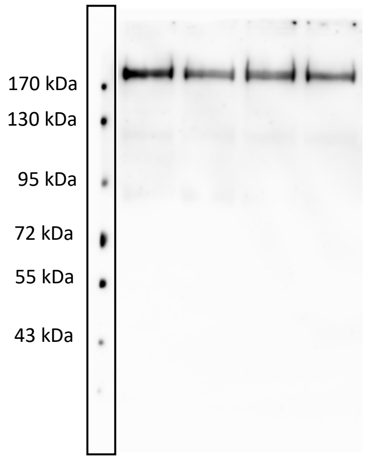

Figure 5F.

αpPKC (~ 82 KDa) **αPKC** (~ 77 KDa)

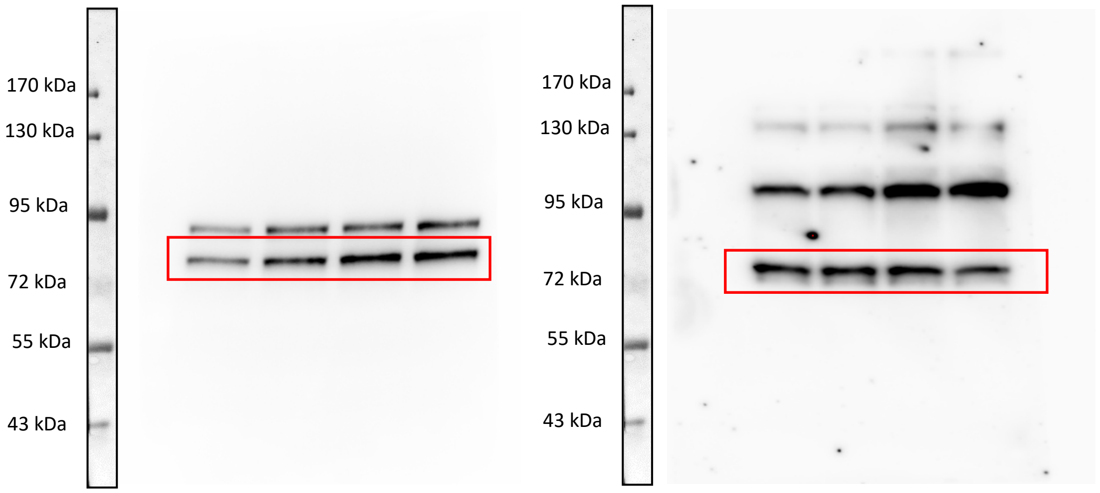

Figure 5K.

αNR2B (~ 180 KDa)

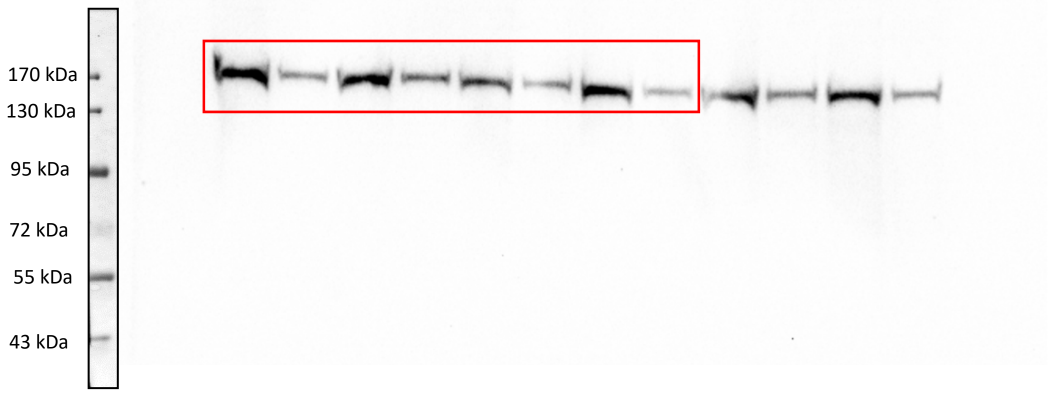

αβ-Actin (~ 42KDa)

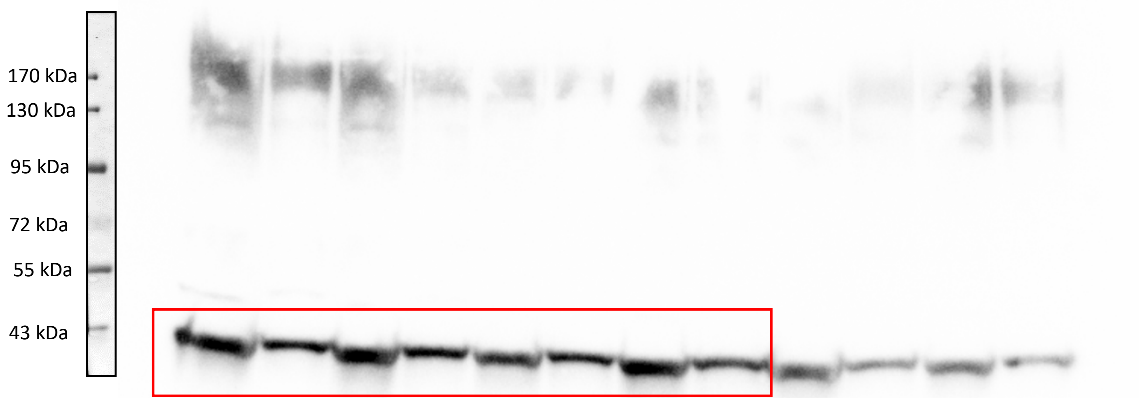

Figure 6A.

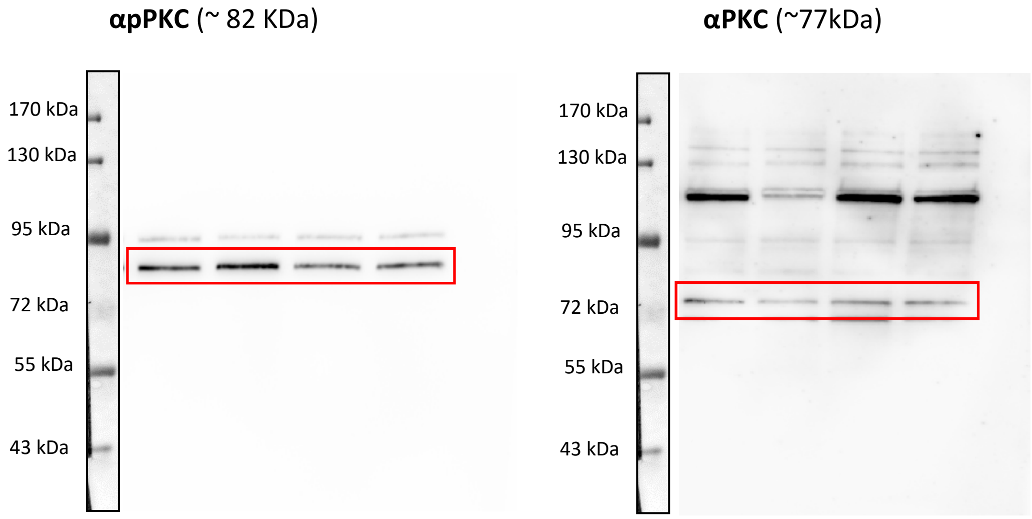

Figure 6B.

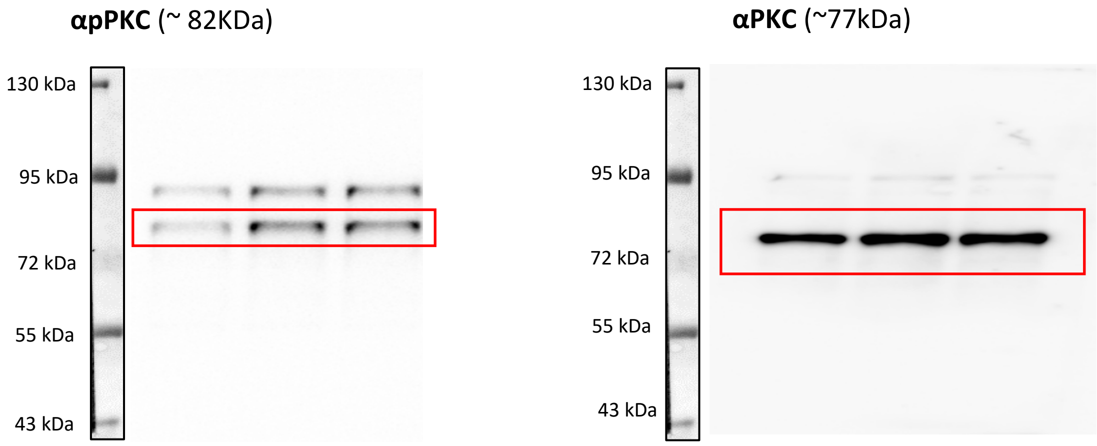

Figure 6F.

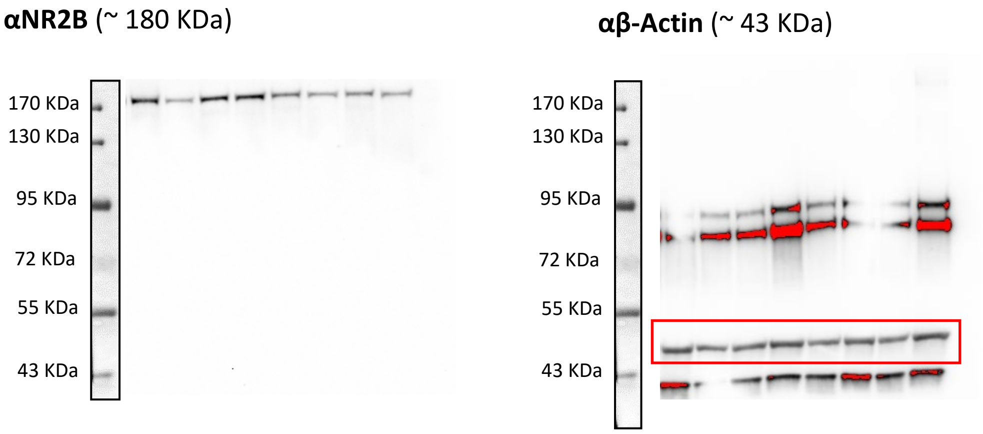

Supplement: Supplementary file 3 — Original Western Blots [file 41419_2022_4687_MOESM3_ESM.pdf]
